# Engineered mitochondria in diseases: mechanisms, strategies, and applications

**DOI:** 10.1038/s41392-024-02081-y

**Published:** 2025-03-03

**Authors:** Mingyang Li, Limin Wu, Haibo Si, Yuangang Wu, Yuan Liu, Yi Zeng, Bin Shen

**Affiliations:** Department of Orthopedics, Orthopedic Research Institute, West China Hospital, Sichuan University, Chengdu, Sichuan Province China

**Keywords:** Genetic engineering, Nanobiotechnology

## Abstract

Mitochondrial diseases represent one of the most prevalent and debilitating categories of hereditary disorders, characterized by significant genetic, biological, and clinical heterogeneity, which has driven the development of the field of engineered mitochondria. With the growing recognition of the pathogenic role of damaged mitochondria in aging, oxidative disorders, inflammatory diseases, and cancer, the application of engineered mitochondria has expanded to those non-hereditary contexts (sometimes referred to as mitochondria-related diseases). Due to their unique non-eukaryotic origins and endosymbiotic relationship, mitochondria are considered highly suitable for gene editing and intercellular transplantation, and remarkable progress has been achieved in two promising therapeutic strategies—mitochondrial gene editing and artificial mitochondrial transfer (collectively referred to as engineered mitochondria in this review) over the past two decades. Here, we provide a comprehensive review of the mechanisms and recent advancements in the development of engineered mitochondria for therapeutic applications, alongside a concise summary of potential clinical implications and supporting evidence from preclinical and clinical studies. Additionally, an emerging and potentially feasible approach involves ex vivo mitochondrial editing, followed by selection and transplantation, which holds the potential to overcome limitations such as reduced in vivo operability and the introduction of allogeneic mitochondrial heterogeneity, thereby broadening the applicability of engineered mitochondria.

## Introduction

Mitochondria are derived from an endosymbiotic progenitor, and a significant portion of the genetic material possessed by the endosymbiont was either lost or integrated into the host genome throughout evolution.^1,2^ This integration ultimately resulted in the emergence of mitochondria as distinct cellular organelles. Due to their endosymbiotic origin, mitochondria possess two distinct membranes: the outer and inner membranes, which enclose the intermembrane gap and the innermost matrix, respectively. The inner membrane creates significant indentations, known as cristae, where most of the composites of the respiratory chain are primarily located. Numerous investigations have shed light on the pivotal role that mitochondria play in energy conversion, biosynthesis, and signal transduction. The view of mitochondria has evolved from adenosine triphosphate (ATP)-synthesizing machines serving in oxidative phosphorylation^3,4^ to multifunctional organelles.^5^ Mitochondria are involved in several pathways and processes, including metabolism (ATP,^3,4^ amino acids,^6,7^ lipids,^8–13^ ascorbate,^14^ carbonate,^15^ reactive oxygen species (ROS),^16,17^ sulfide,^18–20^ the iron–sulfur cluster,^21–23^ tricarboxylic acid and its derivatives,^24–26^ and one-carbon metabolism^27–30^), signal transduction^31^ (sensing,^32–34^ integrating,^35^ and signaling^36,37^), substance transport (calcium,^38–40^ sodium,^38,41^ mitochondrial protein,^42^ mitochondrial-derived vesicles,^43,44^ mitochondrial permeability^45,46^), redox homeostasis,^47,48^ inflammations,^49^ heat production,^50–52^ and cell death regulation.^53^ In addition, mitochondria dynamically recalibrate their biology according to organismal demands and stress, highlighting their multifaceted and complicated traits. Mitochondrial dysfunction and related molecular pathways were reviewed by Zong et al.^54^

**Fig. 1:**
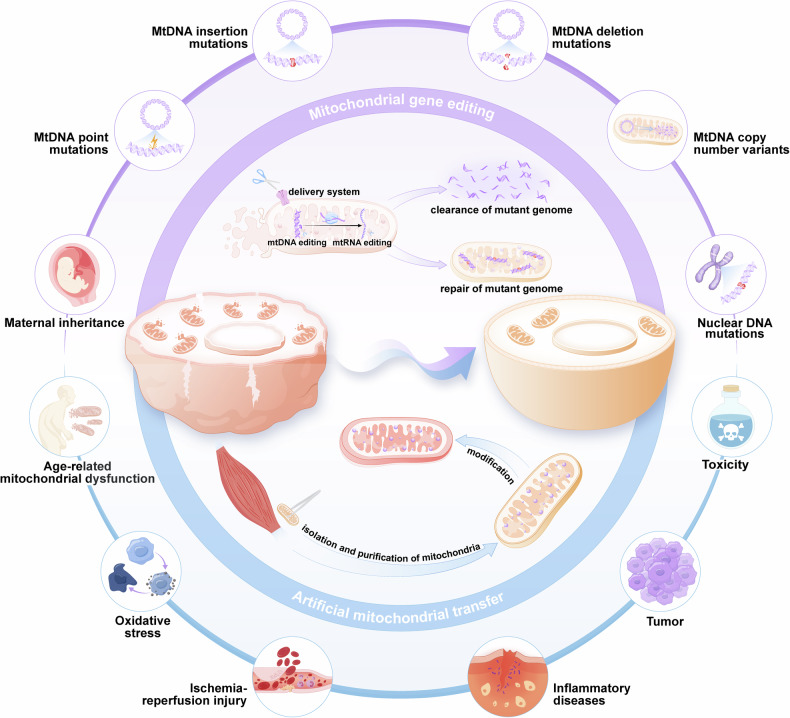
A schematic view of engineered mitochondria in disease therapy. A schematic view of engineered mitochondria in disease therapy. Mitochondrial gene editing precisely addresses abnormalities in mitochondria by clearing or repairing the mutant genome, while artificial mitochondrial transfer restores the activity level of mitochondria by adding functional mitochondria. (Generated by the authors with Adobe Illustrator)

Mitochondria are organelles with uniparental inheritance^55^ and possess their own genome. Mutations in mitochondria can lead to the occurrence of severe diseases,^56,57^ and mitochondrial diseases are a prevailing and devastating category of hereditary genetic disorders that exhibit clinical variability, diagnostic intricacies, and a lack of disease-modifying therapeutic interventions.^58^ Approximately 1000 children born in the United States and 200 children born in the United Kingdom each year are affected by mitochondrial disease.^59^ Recent technological advancements have encouraged the treatment of these disorders through two unique strategies for germline therapy^60–62^: in cases where oocytes or zygotes are susceptible to mitochondrial defects, potential solutions include remedial genome editing or substitution with unaffected mitochondria. In this review, genetically edited mitochondria and isolated mitochondria with or without modifications before transplantation are collectively referred to as “engineered mitochondria”. With the identification of the pathogenic effects of damaged mitochondria in increasingly common diseases, such as aging,^63^ oxidative disorders,^64^ inflammatory diseases,^65^ and cancer,^66^ the application potential of engineered mitochondria has expanded significantly (Fig. 1).

**Fig. 2:**
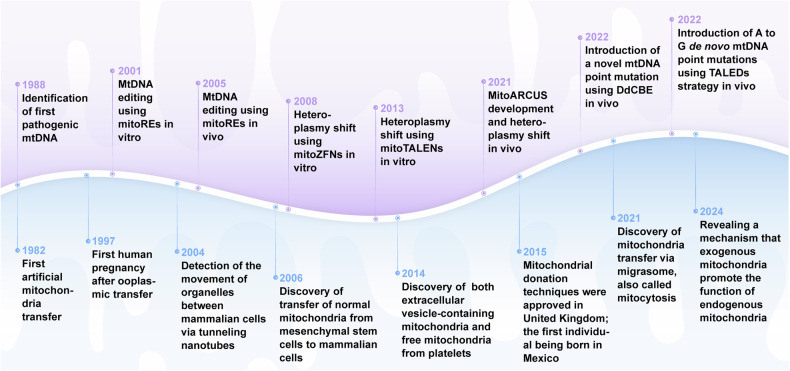
The development of mitochondrial engineering. This milestone timeline provides an overview of the major advancements associated with mitochondrial engineering over the past four decades. Mitochondrial gene editing has progressed from the initial identification of pathogenic mtDNA to the modification of mutants using various nuclease and base editing tools (purple background). Artificial mitochondrial transfer has evolved from the initial achievement of artificial mitochondrial transfer to the initiation of large-scale clinical trials (blue background). DdCBE DddA-derived cytosine base editor, mitoARCUS mitochondrial-targeted meganucleases, mitoREs mitochondrial-targeted restriction endonucleases, mitoTALEN mitochondrial-targeted transcription activator-like effector nucleases, mitoZFNs mitochondrial-targeted zinc finger nucleases, mtDNA mitochondrial DNA, TALEDs TALE-linked deaminases (Generated by the authors with Adobe Illustrator)

In the past decades, encouraging results have been achieved with mitochondrial gene editing and artificial mitochondrial transfer, two distinctive approaches that have developed in parallel. (Fig. 2) The timeline illustrates milestones in the important study of mitochondrial gene editing. In 1988, the identification of the first pathogenic mitochondrial DNA (mtDNA) mutation laid the foundation for understanding mitochondrial diseases.^56^ By 2001, researchers achieved mtDNA editing in vitro using mitochondrial restriction endonucleases (mitoREs).^67^ In 2008, mitochondrial zinc-finger nucleases (mitoZFNs) were used to achieve heteroplasmy shifting, which is a key step in altering the ratio of mutant to wild-type mtDNA.^68^ By 2013, this approach evolved into mitochondrial transcriptional activator-like effector nucleases (mitoTALENs), realizing the heteroplasmy shifting in vitro.^69^ In 2021, the development of mitochondrial-targeted meganucleases (mitoARCUS) enabled heteroplasmy shifting in vivo, a major step towards therapeutic applications.^70^ In 2022, researchers designed a new type of gene editing tools in vivo using a double-stranded DNA cytosine base editor (DdCBE) to enable novel mtDNA point mutations, and a de novo point mutation from A to G using TALE-linked deaminases (TALEDs).^71^ Both technologies further expand the toolkit for precision editing of the mtDNA. These milestones reflect the rapid progress in mitochondrial research and pave the way for potential therapeutic interventions in mitochondrial diseases. The journey of artificial mitochondrial transfer began in 1982 with Clark and Shay^72^ discovering that the transplantation of mitochondria containing antibiotic resistance genes into susceptible cells facilitated their survival in selective media, thereby establishing a novel avenue for research in this field. In 1997, the first human pregnancy after ooplasmic transfer (OT) marked a significant milestone,^73^ demonstrating the potential for mitochondrial manipulation in reproductive medicine. The detection of organelles moving between mammalian cells via tunneling nanotubes in 2004^74^ provided insights into the mechanism of intercellular mitochondrial dynamics. By 2006, the discovery of normal mitochondria transferred from mesenchymal stem cells to mammalian cells^75^ led to numerous subsequent investigations of cellular repair and regeneration via mitochondrial transplantation. In 2014, the identification of extracellular vesicles containing mitochondria and free mitochondria from platelets^76^ suggested a new route of administration. The approval of mitochondrial donation techniques in the United Kingdom in 2015, with the first individual born using this method in Mexico,^77^ represented a major regulatory and clinical breakthrough. In 2021, the first reported mitochondrial transfer via the migrasome,^78^ introduced a novel mechanism for mitochondrial exchange. In 2024, research demonstrated that exogenous mitochondria can promote the function of endogenous mitochondria, providing new perspective on the therapeutic effect of transferred mitochondria.^79,80^

Engineered mitochondria have become a potential solution in regenerative medicine and for treating multiple diseases. In this paper, we comprehensively review the mechanisms and advances in engineered mitochondria for disease therapy and summarize the potential clinical applications and evidence from clinical trials.

## Mechanisms and progress of engineered mitochondria

### Mitochondrial gene editing

#### Mitochondrial transcription and translation

**Fig. 3:**
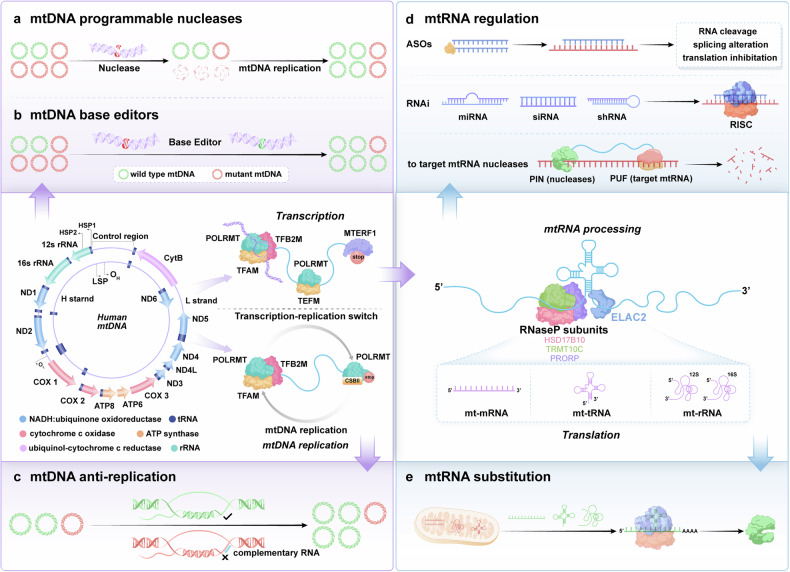
Schematic representation of mitochondrial gene editing strategies. mtDNA replication and transcription initiation: TFAM drives mtDNA replication and transcription, while POLRMT and TFB2M help form the initiation complex at the promoter region. Transcription elongation: After the dissociation of TFAM and TFB2M, POLRMT binds to TEFM for transcription elongation, producing longer transcripts. mtDNA mutant correction: mtDNA mutations are targeted and corrected by nuclease and base editors or suppressed during replication by blockers. Transcription maturation: Transcripts undergo maturation via hydrolytic cleavage by RNase P and ELAC2. **a** mtDNA programmable nucleases: A depiction of programmable nucleases, such as CRISPR-Cas9, designed to target specific sequences in the mitochondrial DNA (mtDNA), enabling precise editing or repair of genetic mutations within the mitochondria. **b** mtDNA base editors: Schematic showing the use of base editors to induce point mutations in mtDNA without causing double-strand breaks, offering a safer alternative for correcting specific genetic mutations. **c** mtDNA anti-replication: Illustration of strategies to inhibit mtDNA replication, including the use of specific blockers that prevent the replication of mutant mtDNA, thus controlling the spread of mutations. **d** mtRNA regulation: Depiction of methods for regulating mitochondrial RNA (mtRNA) expression, including the use of antisense oligonucleotides (ASOs), RNA interference (RNAi), or RNA-targeting nucleases to modulate mitochondrial gene expression. **e** mtRNA substitution: Illustration of the substitution strategy for defective mitochondrial RNAs (mtRNA), such as mt-mRNA, mt-tRNA, and mt-rRNA, to restore normal mitochondrial function by replacing mutated or defective RNA species with functional ones. ASOs antisense oligonucleotides, ELAC2 elaC ribonuclease Z2, HSD17B10 hydroxysteroid (17-beta) dehydrogenase 10, mtDNA mitochondrial DNA, mtRNA mitochondrial RNA, PIN PilT N-terminus, POLRMT mitochondrial DNA-directed RNA polymerase, PRORP proteinaceous RNase P, PUF Pumilio and FBF, RISC RNA-induced silencing complex, RNase P endonuclease P, RNAi RNA interference, shRNA short hairpin RNA, siRNA small interfering RNA, TEFM transcription elongation factor, TFAM mitochondrial transcription factor A, TFB2M mitochondrial transcription factor B2, TRMT10C tRNA methyltransferase 10C (Generated by the authors with Adobe Illustrator)

The human mitochondrial genome is a circular, double-stranded deoxyribonucleic acid (DNA) molecule encoding 2 ribosomal ribonucleic acids (RNAs), 22 transfer RNAs, and 13 proteins involved in mitochondrial oxidative phosphorylation (OXPHOS).^81,82^ The entire mitochondrial genome is transcribed from both strands, resulting in the production of extensive polycistronic transcripts. The classification of these strands is determined by their buoyancy in the density gradients of cesium chloride, resulting in the designation of heavy chains (H) or light chains (L).^83–85^ Initial transcription of mtDNA is driven by mitochondrial DNA-directed RNA polymerase (POLRMT), mitochondrial transcription factor A (TFAM) and mitochondrial transcription factor B2 (TFB2M), and during the elongation stage, transcription elongation factor (TEFM) promotes the processivity of POLRMT, and ultimately mtDNA undergoes bending and base-flipping promoted by mitochondrial termination factor 1 to induce transcription termination. Long polycistronic transcripts require multiple processing steps to form functional RNA species.^86,87^ Most mt-ribosomal ribonucleic acids (mt-rRNAs) and mt-messenger ribonucleic acids (mt-mRNAs) are separated by mt-tRNAs in precursor polycistronic transcripts, and these RNAs undergo different further modifications after being cleaved.^88–91^ In the translation apparatus, mRNAs, transfer ribonucleic acids (tRNAs), and the assembled mitoribosome converge, where translational factors govern the advancement of translation.^92,93^ Initial translation of mRNA matured by mtDNA post-transcriptional processing is driven by the mitochondrial initiation factors mtIF2 and mtIF3, and in the translation elongation phase, codon-anticodon site base pairing is carried out by the mitochondrial elongation factors EFTu (TUFM), EFTs (TSFM), and EFGM (GFM1) in a complex formed with the mitochondrial tRNAs, and ultimately, the stop codon at the A-site triggers the termination of translation and the release of peptides. Currently, the main strategies for mitochondrial gene editing are mtDNA or mitochondrial RNA (mtRNA) engineering (Fig. 3).

#### mtDNA engineering

**Fig. 4:**
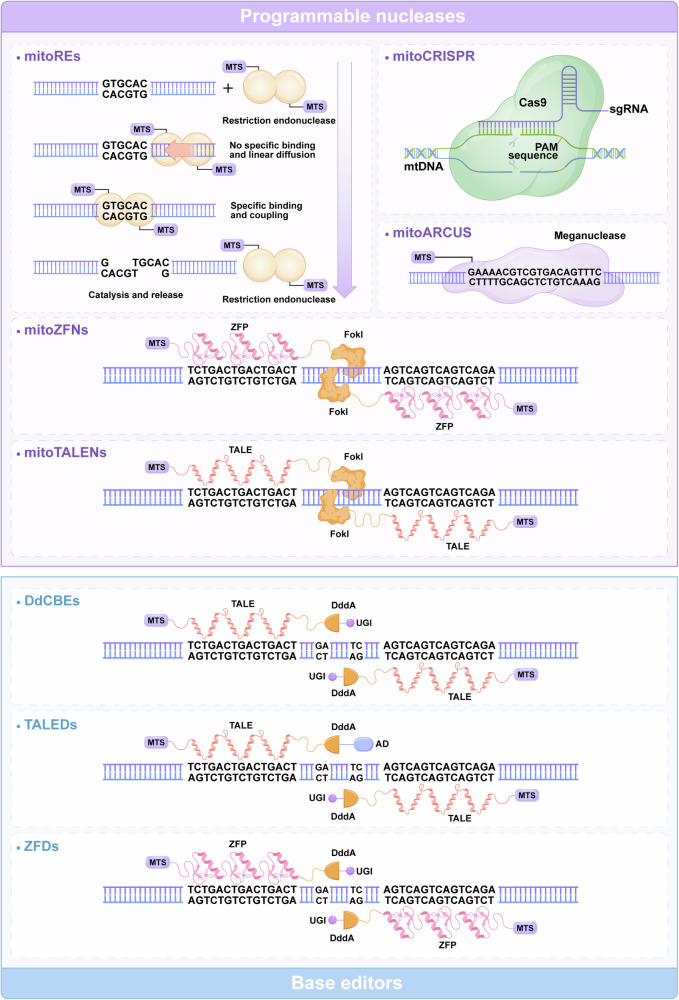
Structure and function of typical mtDNA editing tools. The general architecture of editing tools with programmable nucleases, including mitoREs, mitoARCUS, mtZFN, and mtTALEN (purple background). mitoREs were the first tools used for mtDNA editing, consisting of MTS and endonucleases. mitoARCUS utilizes a significantly modified and simplified I-CreI homing endonuclease. For mitoZFNs and mitoTALENs, the ZFN or TALE domains are utilized to guide the Fok1 restriction endonuclease to target particular gene sequences in mtDNA. mitoCRISPR comprises an sgRNA and a Cas9 endonuclease. The schematic structure of editing tools with base editors, including DdCBEs, TALEDs, and ZFDs (blue background). DdCBEs contain programmable TALE and UGI, enabling the first C–G to T–A conversion. TALEDs utilize adenine deaminase TadA8e and DddA, promoting the conversion of A–G bases in mtDNA. ZFDs consisting of the zinc finger DNA-binding protein DddA and UGI, catalyze C-to-T conversion. AD adenosine deaminase; DdCBEs DddA-derived cytosine base editors; mitoARCUS mitochondrial-targeted meganuclease; mitoCRISPR mitochondrial-targeted clustered regularly interspaced short palindromic repeats; mitoREs mitochondrial-targeted restriction endonucleases; mitoTALENs mitochondrial-targeted transcription activator-like effector nucleases; mitoZFNs mitochondrial-targeted zinc finger nucleases; mtDNA mitochondrial DNA; MTS mitochondrial targeting sequence; PAM protospacer-adjacent motif; sgRNA guide RNA; TALEDs TALE-linked deaminases; UGI uracil glycosylase inhibitor; ZFDs zinc finger deaminases; ZFP zinc finger proteins (Generated by the authors with Adobe Illustrator)

The correlation between the quality of mtDNA and the function of mitochondria is considerable, and mutations in mtDNA could result in hereditary mitochondrial diseases.^94,95^ Unfortunately, the mutation susceptibility of mtDNA outweighs that of nuclear DNA for reasons including the proximity of mtDNA to the OXPHOS system,^96–98^ the limited number of DNA repair mechanisms inside mitochondria, the absence of canonical protection proteins, and the high replication and mistake rate.^99–101^ Pathogenic mutations in mtDNA lead to one of two states: heteroplasmy or homoplasmy. Heteroplasmic mutation is characterized by the coexistence of pathogenic mtDNA mutations with unaffected mtDNA molecules. Homoplasmic mutation refers to the condition in which cells only possess mutant mtDNA (devoid of wild-type mtDNA). In recent years, mitochondrial gene editing has substantially advanced the use of anti-replicative agents, programmable nucleases, and base editors.^102–104^ For heteroplasmic mutations, strategies include shifting the heteroplasmy ratio by selectively degrading mutant mtDNA or enhancing the replication of wild-type mtDNA.^67^ Restriction enzymes or programmable nucleases could shift the ratio of heteroplasmic mutations, while these strategies are unable to repair homoplasmic mutations.^105^ For homoplasmic mutations, techniques such as allotropic expression are being explored.^96^ The base editors have the potential to correct both heteroplasmic and homoplasmic mtDNA mutation^71^ (Fig. 4).

##### mtDNA engineering with anti-replicative agents

The targeted disruption of pathogenic mtDNA replication can be achieved through specific small molecules capable of annealing with mutant sites. The first reported approach for shifting mtDNA heteroplasmy involves disrupting mtDNA replication by using peptide nucleic acid oligomers (PNAs).^106^ These small molecules with nucleobases linked to an achiral peptide backbone exhibit higher affinity than equivalent oligodeoxynucleotides when binding to single-stranded complementary DNA.^107^ However, delivering PNAs across the inner mitochondrial membrane is challenging.^107^ Another approach was developed using anti-replicative oligoribonucleotides that can be as efficient as synthetic PNAs.^107^ Because 5S rRNA is partially imported into human mitochondria,^108^ recombinant 5S rRNA molecules, which use 5S rRNA as a vector to deliver anti-replicative oligoribonucleotides, were specifically developed to regulate mtDNA heteroplasmy.^109^ However, these anti-replication agents were developed in vitro replication under physiological conditions, no ideal experimental results have been observed on either cell culture or isolated mitochondria. Mitochondria are highly dynamic, undergoing fusion and fission, and there is currently no consensus on the molecular mechanisms of nucleic acid trafficking within mitochondria or its potential functions. This gap in understanding complicates the stability of changes introduced by anti-replicative strategies.

##### mtDNA engineering with programmable nucleases

The presence of heteroplasmic characteristics in most mitochondrial disorders, the swift breakdown of mtDNA molecules that contain double-strand breaks (DSBs), and the precise regulation of mtDNA copy number give rise to a distinctive therapeutic concept known as “heteroplasmy shifting.” The use of programmable nucleases is the most widespread and efficient method.^110–114^ This approach relies on the specific cleavage of endonucleases at mutation sites in mtDNA, with the aim of degrading the entire pathogenic mtDNA.^115^ The absence of a nuclease recognition site in wild-type mtDNA results in its preservation, enabling the progressive replacement of mutant copies and subsequent restoration of mitochondrial function by wild-type mtDNA.^69,116,117^ mitoREs, mitoTALENs, mitoZFNs, and mitoARCUS are comprehensively described in the subsequent sections.

**mitoCRISPR**. The clustered regularly interspaced short palindromic repeats (CRISPR)/CRISPR-associated protein 9 (Cas9) system for nuclear gene editing was initially introduced in 2013 and can achieve sequence-specific DNA recognition and cleavage.^118,119^ The bacterial immune system incorporates CRISPR/Cas9 as a mechanism to safeguard cells against exogenous DNA.^119–124^ The system comprises a single guide RNA (sgRNA) that selectively identifies the gene sequence and a Cas9 endonuclease that produces DSBs at the adjacent motif.^125–130^ CRISPR/Cas9 has been successfully employed to specifically target the Cox1 and Cox3 loci within mtDNA, disrupting the mitochondrial membrane and subsequently inhibiting cell growth.^131,132^ Researchers have made progress in the targeted cleavage of mtDNA in HEK293T cells and zebrafish.^133,134^

However, this method is unsuitable for modifying the mammalian mitochondrial genome. Introducing sgRNA into human mitochondria remains challenging, limiting its widespread acceptance.^135,136^ The current lack of resolution in addressing this limitation highlights the inefficiency of this approach. Several researchers have made noteworthy efforts in the delivery of sgRNA; nevertheless, none of the studies conducted thus far have resulted in a shift in heteroplasmy, and this approach has consequently failed to achieve widespread acceptance within the research community.^133,134,137^ Until recently, the application of CRISPR-based systems for mtDNA manipulation has not been consistent.^135,138–140^

**mitoREs**. mitoREs were an early tool used to create site-specific DNA DSBs to cleave gene sequences. Pathogenic mtDNA variants can lead to the development of distinct restriction sites, and these tools can efficiently eliminate pathogenic mtDNA and facilitate heteroplasmy shifting. Certain nucleases, such as mitoPstI and mitoApaLI, can transport mitochondrial target signals (MTSs) via the mitochondrial transport machinery.^67,141^ Once they enter the mitochondria, mitoREs specifically target restriction sites in the mutant, causing targeted breaks that lead to mtDNA elimination and heteroplasmy shifting. These tools have shown promising results in in vitro models of primary mitochondrial diseases and in vivo models, such as mice with asymptomatic mtDNA heteroplasmy.^141,142^ In addition, human cybrid cell lines have been used to assess the effectiveness of mitoREs. The application of mitoREs to these cells decreased the extent of the mutation and improved physiological processes, such as oxidative phosphorylation activity and ATP production.^143–145^ Although these tools are effective in gene editing for mtDNA, these approaches have strict sequence limitations, making them the least versatile option in the mitochondrial gene editing toolbox.

**mitoZFNs and mitoTALENs**. mitoZFNs and mitoTALENs are modified nucleases that specifically target mitochondria. ZFN or transcription activator-like effector (TALE) domains are utilized to guide the Fok1 restriction endonuclease to target particular gene sequences in mtDNA. This approach overcomes the constraint of mitoREs, which are limited by the number of unique recognition sites for restriction endonucleases in the mtDNA variant.^69,115,146,147^ ZFN dimers cleave mtDNA at precise locations. These ZFNs comprise a sequence-independent endonuclease and a DNA-specific recognition domain.^148^ The homodimerization of FokI domains in both ZFN and TALEN monomers necessitates a high degree of closeness, leading to DSBs.^149^ mitoZFNs and mitoTALENs were subsequently created using MTS technology.^69,150,151^ Nevertheless, the practical application of mitoTALENs is restricted due to their substantial dimensions and heterodimeric composition, necessitating the packaging of each monomeric constituent into an independent viral vector.^152^

mitoZFNs have been evaluated in vivo using *m.5024C* > *T* tRNA-Ala mice. mitoZFNs were packaged with the cardiotropic Adeno-associated viruses (AAV) and administered through intravenous injection to male mice aged 2–8 months.^153^ The mice were euthanized 65 days after injection and exhibited heteroplasmy in the heart. The levels of tRNA-Ala, lactate, and pyruvate were restored, re-establishing the normal activity of cardiac mitochondria.^68,154^ In addition, mitoTALENs have been successfully used to detect mitochondrial alterations in mouse embryos.^155^ In the heteroplasmic NZB/BALB mouse model, the number of NZB mitochondrial genomes was selectively reduced in M-II oocytes utilizing mitoTALENs, preventing NZB mitochondrial genome transmission to the next generation. Following the successful introduction of the pathogenic human *m.14459G* > *A* and *m.T9176T* > *C* mutations, mouse oocytes were targeted using a relevant set of mitoTALENs in similar attempts. After mRNA microinjection and mitoTALEN expression, total mtDNA levels were not recovered because oocytes and preimplantation embryos cannot replicate mtDNA.^156,157^ Therefore, given the elevated mutant load of oocytes, the possibility of producing nonviable embryos following a mitonuclease treatment must be considered.

**mitoARCUS**. mitoARCUS was created to overcome the obstacles presented by the previously mentioned tools.^70^ ARCUS gene editing technology, created by Precision Biosciences, utilizes a significantly modified and simplified I-CreI homing endonuclease. mitoARCUS can be adjusted to target and cleave nearly any base sequence using computational evolution methods.^158^ The gene editing technique was evaluated in the MT-TA *m.5024C* > *T* animal model, known for reduced tRNA-Ala levels and mitochondrial cardiomyopathy.^70^ MitoARCUS packed with AAV9 was administered via retro-orbital injection to mice aged 2.5 or 6 weeks, and the mice were euthanized 6–24 weeks after the injection.^159^ The heteroplasmy levels were evaluated in various organs, including the heart, kidney, liver, and spleen. The most significant change occurred in the liver, possibly because of the pronounced hepatic tropism exhibited by AAV9. Moreover, the levels of tRNA-Ala in the same organ were effectively restored.

##### mtDNA engineering with base editors

Mito-nucleases can eliminate mutant mtDNA but cannot repair defective genomes. Therefore, these techniques are ineffective in rescuing pathogenic, homoplasmic mutations.^160^ This situation requires a distinct method to alter mitochondrial DNA.

Mok et al. discovered an interbacterial toxin, DddA, that catalyzes the deamination of cytidines within dsDNA; engineered split-DddA halves were developed that remained inactive until they interacted on target DNA.^62^ Several mitochondrial base editors have been developed based on this tool, including DdCBEs, TALEDs, and zinc-finger deaminases (ZFDs).

**DdCBEs**. Mok et al. utilized DddA, a double-stranded DNA deaminase of *Burkholderia cepacia* origin, and fused and assembled it with a programmable TALE and uracil glycosylase inhibitor (UGI) to generate cytosine base editors of DddA origin, enabling the first specific and efficient C–G to T–A conversion in mtDNA.^62^ Since DddA is a bacterial enzyme that is toxic to mammalian cells, researchers split DddA into two halves and fused each to a mitochondrion-targeted TALE array, thus ensuring that the two halves regained catalytic activity only when bound together at the targeted gene regions. The dU formed after cytosine deamination is excised by endogenous uracil glycosylase (UNG). Two TALEs are fused to UGI to prevent dU from being excised, and after DNA replication or repair, the final base-pair transition from C–G to T–A is achieved. In addition, DdCBEs have been utilized to construct disease models in human cells with mtDNA mutations, resulting in changes in respiration rates and oxidative phosphorylation. DdCBEs can decrease the proportion of mutant mtDNA without affecting the total number of copies.^62^ Editing efficiencies vary from 5% to 50%, which has implications for researching mitochondrial diseases and treating them instead of deleting mtDNA copies resulting from specific nuclease activity.^62,161^

DdCBEs have been effectively introduced into postnatal mice.^162^ Perdo et al. used AAV9.45 to deliver DdCBEs into the heart tissue of 8-week-old and 1-day-old mice by inducing two specific edits at the MT-Nd3 site: *m.9576G* > *A* and *m.9577G* > *A*.^163^ DdCBE-specific edits were detected in both adult and newborn mice after injection. However, significant mitochondrial off-target editing was observed at any time after treatment.^164,165^ DdCBEs have been used in rats to generate mutations equivalent to the human pathogenic *m.8363G* > *A* and *m.14710G* > *A* mutations to investigate whether DdCBEs can edit mtDNA in vivo and construct animal models that mimic human mtDNA mutant diseases.^163,166–169^ Lee et al. microinjected DdCBE-encoding mRNA into mouse embryos to introduce the *m.12336C* > *T* nonsense mutation into the mitochondrial Nd5 gene, mimicking the Leber hereditary optic neuropathy (LHON), and Leigh syndrome in human diseases.^170^ Shen et al. microinjection of DdCBE-encoding mRNA into zebrafish to generate the *m.4247G* > *A*, *m.14076G* > *A*, and *m.8892G* > *A*, stimulating *m.3733G* > *A*, *m.13513G* > *A*, and *m.8363G* > *A* mutations in human mtDNA to creat various disease models.^166^ These efforts have provided important animal models for studying clinical gene therapy options and the pathogenic mechanisms of mitochondrial diseases.

**TALEDs**. The first mitochondrial base editor to achieve A-to-G base editing of human mtDNA used transcriptional activator-like effector-linked deaminases called TALEDs, which were developed after the discovery of DdCBEs.^71^ Each of the three primary parts of TALEDs has a distinct purpose. TALE, a DNA binding protein that targets particular DNA sequences, is the first component. TALE can attach to particular mitochondrial DNA sequences via mitochondrial localization signals once they enter the mitochondria. The adenine deaminase TadA8e, which promotes the conversion of A–G bases in mtDNA, is involved in the second pathway. DddA, the third component, facilitates the easier editing of mtDNA by TadA8e.^171^ This base editor targets any A within the spacer region, regardless of the context or strand, with a preference for those located in the central part rather than at the edges. This innovative tool has been evaluated in various human cell lines as a proof-of-concept, successfully editing 17 different target regions within the mitochondrial genome. Although most of the changes did not exhibit any mitochondrial characteristics, the editing of the gene *MT-RNR2* resulted in a mutation that conferred chloramphenicol resistance.^71^ Moreover, when this medication was introduced, the modified cell line ultimately became homoplasmic for the mutation.

**ZFDs**. ZFDs, consisting of the zinc finger DNA-binding protein DddA and UGI, catalyze C-to-T conversion. ZFDs combine MTS and nuclear output signal sequences, forming a functional ZFD system, achieving base editing efficiencies of 60% in nuclear DNA and 30% in mtDNA.^172^ Zinc finger arrays (2 × 0.3–0.6 kb) in ZFDs are compact and, therefore, smaller in size than the TALE arrays (2 × 1.7–2 kb) in DdCBE. Additionally, compared to TALEs, zinc fingers lack bulky structural domains at both the C-terminal and N-terminal ends, making them somewhat easier to use: a split DddA can be fused to either end of the zinc finger arrays. Direct delivery of the purified ZFD protein into human cells via electroporation resulted in up to 27% targeted C-to-T conversion. These properties make ZFDs an ideal nuclear and organelle DNA base editing platform.^173^

Collectively, mitoCRISPR offers simplicity of design, high flexibility, and efficiency in creating targeted DSBs, making it a powerful tool for various genetic modifications. However, it has the highest risk of off-target effects and faces significant delivery challenges due to the lack of effective delivery systems. mitoREs are highly specific for target sequences, which reduces off-target effects, but they have a limited targeting scope and can only recognize a narrow range of sequences. mitoTALENs and mitoZFNs combine high specificity with a broader targeting scope compared to mitoREs, but their construction is complex and difficult in packaging. mitoARCUS provides high specificity and minimal off-target effects, but similar to mitoTALENs and mitoZFNs, it involves challenging engineering processes. DdCBEs enable precise base editing without introducing DSBs, while they have a limited targeting range and can be difficult to deliver effectively. TALED offers precise editing with minimal off-target effects, but its application is limited by the complexity of TALEN design and delivery issues. ZFDs are highly specific and versatile, but their engineering is complex, and they have a higher potential for off-target effects compared to some other tools. Each tool’s relevance to mitochondrial diseases depends on balancing these strengths and weaknesses, with delivery and specificity being critical factors for therapeutic.

Targeted editing of mtDNA is now possible through the specific removal of mtDNA using nucleases. The use of artificial nucleases allows for more versatile design and application. mtDNA base editors may be used in heteroplasmy and homoplasmy situations and can create new mtDNA mutations at particular locations. Thus, these editors are a valuable tool for developing mtDNA mutation models, filling the gap left by existing models. Table 1 outlines the features of the currently available primary mitochondrial DNA editing tools.^67,70,71,115,134,150,165,172^

**Table 1:** Mitochondrial DNA editing technologies

Editing tools	Major components	Size	Advantages	Disadvantages	Editing effect	Ref
mitoCRISPR	Cas9 + sgRNA	Large (two components)	Easy to design High flexibility	High off-targeting effect Lack of effective delivery	Knocked into or down specific mtDNA	^134^
mitoREs	MTS + RE	Small (Homodimer or monomer)	Low off-targeting effect	Engineering is challenging Limited targeting scope	Elimination of specific mtDNA	^67^
mitoZFNs	MTS + ZF+FokI	Relatively large (Heterodimer)	RNA-free	Difficulties in packaging	Elimination of specific mtDNA	^150^
mitoTALENs	MTS + TALE+FokI	Large (Heterodimer)	RNA-free	Delivery challenge	Elimination of specific mtDNA	^115^
mitoARCUS	MTS+meganuclease	Small (Homodimer or monomer)	Recognize changes in individual bases	Engineering is challenging Limited targeting scope	Elimination of specific mtDNA	^70^
DdCBE	MTS + TALE+DddA+UGI	Large (Heterodimer)	High flexibility	Possible off-targeting effect	mtDNA point mutation (C-to-T)	^165^
TALED	MTS + TALE+DddA+TadA8e	Large (Heterodimer or monomer)	High flexibility	Bystander editing	mtDNA point mutation (A-to-G)	^71^
ZFDs	MTS + ZF+DddA+UGI	Relatively large (Heterodimer)	High flexibility and low immunogenicity customizable binding	Engineering is challenging	mtDNA point mutation (C-to-T)	^172^

*AD* adenosine deaminase, *Cas9* CRISPR-associated protein 9, *DdCBE* DddA-derived cytosine base editor, *mitoARCUS* mitochondrial-targeted meganuclease, *mitoCRISPR* mitochondrial-targeted clustered regularly interspaced short palindromic repeats, *mitoREs* mitochondrial-targeted restriction endonucleases, *mitoTALEN* mitochondrial-targeted transcription activator-like effector nuclease, *mitoZFNs* mitochondrial-targeted zinc finger nucleases, *MTS* mitochondrial targeting sequence, *PAM* protospacer-adjacent motif, *sgRNA* guide RNA, *TALED* TALE-linked deaminase, *UG*I uracil glycosylase inhibitor, *ZFD* zinc finger deaminase, *ZFP* zinc finger proteins

#### mtRNA engineering

The frequency of RNA synthesis is comparable to that of RNA encoded from a single strand of mtDNA.^83,174–177^ Nonetheless, gene expression is strictly regulated by posttranscriptional processing and translation.^178^ With the advances in mitochondrial RNA engineering,^179–183^ strategies at various genetic levels, such as mtRNA processing, stability, or transcript translation, have become promising for treating mitochondrial diseases^178,184^ (Fig. 3).

##### mtRNA engineering with nucleic acid tools

Various nucleic acid tools specifically target RNA molecules to regulate gene expression by influencing transcript processing, stability, or translation.^185–188^ Nucleic acid tools provide exceptional specificity and the potential for quick and efficient manufacturing.^189,190^ mRNA-based medicines and alternative wild-type mtRNAs can serve as effective treatments to address faulty mtRNAs and proteins.^107,191,192^

**Antisense oligonucleotides (ASOs)**. ASOs have been successfully transported into mitochondria for several decades and have been demonstrated to function in mitochondria.^193,194^ In particular, new transport tools based on ASOs improve the safety and efficiency of mitochondrial gene silencing.^195^ Cruz-Zaragoza et al. developed a new ASO system, morpholino oligonucleotides (MOs), to selectively and efficiently inhibit mtRNA translation.^196^ Researchers have further utilized the mitochondrial protein Jac1 as a vector to facilitate the import of MOs into mitochondria given the protein’s small size, simple folding, solubility, and the presence of only one cysteine.^197^

**mitoRNAi**. RNA interference (RNAi) can silence genes through the RNA-induced silencing complex using various molecules, such as small interfering RNAs (siRNAs), microRNAs (miRNAs), short hairpin RNAs (shRNAs), long non-coding RNAs (lncRNAs), and circular RNAs (circRNAs). These molecules target specific mRNAs to reduce abnormal protein levels or regulate noncoding RNAs (ncRNAs) associated with various diseases.^198–201^

Although the intrinsic mitochondrial RNAi mechanism remains unknown, targeting mitochondrial RNA is therapeutically promising, given the existing understanding of mitochondrial biology and pathologies.^190,202–204^ Multiple therapeutic RNAi agents have been shown to suppress the translation of their target mitochondrial mRNAs.

miRNAs are present and active in mitochondria.^205,206^ In 2004, Zhang et al. discovered that miRNAs and their effector protein Ago2 are present in mitochondria and increase mitochondrial gene expression during muscle development, indicating the ability of miRNAs to penetrate mitochondria.^198^ In addition, siRNAs designed using Ago2-binding peaks might selectively target matching mtDNA-encoded transcripts in an Ago2-dependent manner. Mitochondrial function is greatly impacted by knocking down mitochondria-encoding subunits with mitoRNAi. These RNAi agents offer significant potential for modulating mitochondrial gene expression and improving mitochondrial function. (Table 2) Their relevance to mitochondrial status lies in their ability to specifically target and regulate mitochondrial mRNAs, offering therapeutic benefits in various mitochondrial diseases. The observed effects on mitochondrial function, such as improved respiration, reduced oxidative stress, and enhanced mitochondrial biogenesis, highlight their potential as promising therapeutic tools. This approach might offer a possible remedy for disorders resulting from mitochondrial DNA mutations.^207^

**Table 2:** Representative therapeutic RNAi agents

Tools	Target	Mechanism of action	Example	Ref
siRNA	Specific mitochondrial mRNAs	Binding to complementary sequences in mitochondrial mRNAs, leading to their degradation and preventing translation.	siRNA targeting ND1, COXI, COXII, and COXIII in HEK293T cells	^207^
shRNA	Specific mitochondrial mRNAs	Processed into siRNA-like molecules that bind to and promote the degradation of specific mitochondrial mRNAs.	shRNA targeting TFAM in SW480 and Caco-2 cells	^509^
miRNA	Specific mitochondrial mRNAs	Binding to target sites on mitochondrial mRNAs, leading to translational repression or degradation	miRNA-2392 regulating oxidative phosphorylation and glycolysis in tongue squamous cell carcinoma	^510^
lncRNA	Mitochondrial gene expression regulators	Interacting with mitochondrial mRNAs or proteins involved in mitochondrial transcription and translation	lncRNA targeting cytochrome B ameliorated mtDNA damage in retinal endothelial cells	^511^

*lncRNA* long non-coding RNA, *miRNA* microRNA, shRNA short hairpin RNA, *siRNA* small interfering RNA

**RNA supplementation**. Another therapeutic nucleic acid tool is replacing or compensating for defective RNAs in the mitochondrial matrix using RNA supplements, including functional tRNAs, rRNAs, and mRNAs. Researchers have effectively restored mitochondrial respiration rates in patient-derived fibroblasts after targeting and delivering pre-tRNA^Phe^ and 12S rRNA to mitochondria with the m.G625A mutation in the tRNA^Phe^ gene and the *m.A1555G* mutation in the 12S rRNA gene.^208,209^ Additionally, the delivery of coding RNAs translated into one or more proteins to the mitochondrial matrix is an effective therapeutic strategy for treating mitochondrial diseases. For example, the delivery of polyribonucleic acid with eight in vitro-assembled RNA import complex (RIC) subunits to the mitochondria of Kearns–Sayre syndrome mice effectively restored mitochondrial respiratory efficiency and quality.^210^ The introduction of mRNA containing the human *COX2* gene into the mitochondria of mouse embryonic fibroblasts resulted in the successful production of functional *COX2* protein in the matrix.^192^ Yamada et al. reported that introducing mRNA for the *ND3* gene into the mitochondria of fibroblasts from Leigh syndrome patients with the *m.T10158C* mutation in the *ND3* gene also improved the mitochondrial respiration rate.^211,212^

##### mtRNA engineering with protein-based tools

In addition to nucleic acid tools, many synthetic protein tools have been designed to act on mitochondrial transcriptional processes, including coordinated RNA processing, polyadenylation, maturation, and RNA attenuation, by selectively binding to specific RNA targets within the mitochondria. Artificial site-specific RNA endonucleases (ASREs) contain a customizable, sequence-specific RNA-binding structural domain with an RNA nuclease structural domain.^213^ The structural domain of the Pumilio and FBF (PUF) targets an 8-nucleotide RNA sequence, and the PilT N-terminus (PIN) structural domain of SMG6 nonspecifically cleaves RNAs. PUF–PIN-based ASREs specifically repress the transcription of mitochondrial target genes.^214^ ASREs bind to the N-terminal targeting peptide of ornithine transcarbamylase to form mitoASREs, facilitating protein-targeted import into diseased mitochondria. Choudhury et al. designed mitoASREs with *dehydrogenase (ubiquinone) Fe-S protein 4* (*NADH) dehydrogenase subunit 5* (*MT-ND5*) as a target gene, and the transcript levels of *MT-ND5* decreased by 20–30% after these mitoASREs were transfected into human cells.^213^

#### Mitochondrial gene delivery

Mitochondria have their own genetic system but rely heavily on importing several macromolecules encoded by the nucleus to maintain gene expression.^215,216^ Certain noncoding genes from the nuclear genome are partly transferred to the mitochondria in all eukaryotes. This phenomenon raises questions about the functional importance of these genes and the exact processes of transportation.^217,218^ Understanding gene import phenomena relies heavily on the effectiveness of the analytic tools used to identify and describe imported genes.^219^ Recent methodological advances using specifically designed in situ and interactomic techniques show potential for addressing many of these problems. Mitochondrial gene import requires three main components: import determinants within the gene, a mechanism to redirect RNA to the mitochondria, and a pathway to transport nucleic acid across the mitochondrial membrane.^220–225^

Introducing nucleic acid therapeutic tools into mitochondria by breaking through complex barriers, such as bilayer membrane structures, is necessary to realize gene therapy for mitochondrial diseases.^226–228^ Research on mitochondrial gene delivery has a four–decade–long history. Therapeutic nucleic acid medications are susceptible to degradation by nucleases in the blood and are quickly eliminated by the reticuloendothelial system and kidneys,^229,230^ impeding their efficient accumulation in mitochondria.^231–233^ Therefore, appropriate carriers are required to safeguard therapeutic nucleic acids and transport them to mitochondrial targets.^234–238^ Progress in drug delivery technology has allowed the containment of different cargos, including tiny pharmaceuticals, nucleic acids, and proteins, and the precise targeting of certain tissues and cell types to improve delivery effectiveness.^239–242^ In recent years, mitochondrial gene delivery has been developed to control the intracellular transportation of delivery vehicles to target specific organelles.^243–246^ Targeting the appropriate organelle can improve treatment effectiveness and reduce negative side effects.^247,248^ The essential activities of mitochondria rely on crucial proteins expressed by both nuclear and mitochondrial genes. Thus, the advancement of gene delivery methods has been a focal point of research, leading to the creation of several tools.^236,249–252^

##### Physical approaches

Physical gene delivery is the direct transfer of therapeutic nucleic acids into the cytoplasm or nucleus through membrane penetration without the use of chemicals or other carrier molecules, such as viruses. Microinjection directly delivers exogenous genes into cells through fine glass microtubes. In addition, hydrodynamic delivery increases hydrodynamic pressure by rapidly pushing a large volume of solution into the bloodstream, temporarily enhancing the permeability of the cell and allowing therapeutic nucleic acid tools to enter the cytoplasm, which they would not normally be able to do when unable to cross the cell membrane. Yasuzaki et al. showed that plasmid DNA could be injected intravenously into rat liver mitochondria by hydrodynamic injection.^253,254^ In contrast to chemical and biological processes, physical approaches do not cause toxicity when linked to carrier molecules. However, physical agents are equally dispersed in the cytoplasm and reach the mitochondrial matrix randomly. With the evolution of technology, the ability to deliver genes that are truly targeted to mitochondria via physical approaches will likely emerge.

##### Chemical approaches

Most methods developed for mitochondrial gene delivery are chemical. Physical methods enhance the absorption of nucleic acid drugs by cells through physical forces, thereby improving gene delivery efficiency, whereas chemical methods utilize the hydrophobic and negatively charged nature of mitochondrial membranes to deliver nucleic acid drugs through special chemical interactions with mitochondrial membranes. Nanotechnology has become a significant focus of chemical approach research with the advancement of the nanoscience era.^255–257^ Lipid-based, polymer-based, mitochondrial-targeted peptides, and inorganic-based nanoparticles have facilitated the creation of innovative and unique breakthroughs in mitochondrial gene delivery, which have been adapted to reduce immunogenicity, enhance safety profiles, and the flexibility to load gene materials.^258,259^

**Lipophilic cations**. The use of lipophilic cations as ligand molecules, such as triphenylphosphine cation (TPP^+^), which has high lipophilicity and a large ionic radius, effectively reduces the activation energy required for transmembrane transport and allows the effective targeting of genes to mitochondria. Compared with hydrophilic cations (e.g., Na^+^), lipophilic cations (e.g., TPP^+^) can accelerate the transport of bioactive molecules across the mitochondrial membrane by as much as 10^7^–10^8^ times. Faria et al. coupled TPP with Polyethylene Glycol (PEG)–Polyethylenimine (PEI) to form PEG–PEI–TPP. After complexing pDNA for targeted mitochondrial delivery, PEG–PEI–TPP significantly increased the efficiency of *ND4* gene and protein expression.^260^

Dequinium chloride (DQA) is a bicationic compound comprising two symmetric molecules, and the vesicle-like aggregates formed by DQA with diameters ranging from 70 to 700 nm are known as DQAsomes, which are recognized as effective carriers that target mitochondria.^261–263^ DQAsomes containing pDNA expressing green fluorescent protein (GFP) stimulate mitochondrial gene expression in cultivated cells.^264^ The transfection efficacy of DQAsomes in the mitochondria was only 5%. Choi and colleagues combined DQA with additional lipids, 1,2-Dioleoyl-3-trimethylammonium-propane (DOTAP) and dioleoylphosphatidylethanolamine (DOPE), to enhance the functionalization of DQAsomes.^265^ Compared with the cellular uptake and endosomal escape capabilities of DQAsomes alone, those of DQA, in combination with DOTAP/DOPE, were enhanced, increasing mitochondrial gene expression.

Previous research has revealed that a carrier based on octaarginine (R8)-modified liposomes, referred to as MITO-Porters, has strong mitochondrial fusion activity.^266–268^ Similar to conventional cationic liposomes, these particles can be internalized by macropinocytosis rather than clathrin-mediated endocytosis.^269^

**Dendrimers**. Dendrimers are nanoscale polymers with a dendritic backbone and a spherical shape with many active functional groups attached to the ends of the dendritic branches. Drugs can be either encapsulated in the internal cavities of the dendrimer backbone or chemically coupled to the functional groups on the surface of the dendrimer.^270–273^ Dendrimers possess a dynamic structure and synthesis process that allows them to be easily tailored for specific applications. Dendrimers have a core-shell structure that enables the creation of branch points during synthesis. These branch points can either be encapsulated in the internal cavities of the dendrimer backbone or chemically coupled to the functional groups on the dendrimer surface.^274,275^ Wang et al. investigated the transport of mitochondrial genes utilizing dendrimers as carriers. They evaluated the generation 5 polyamidoamine (G5-PAMAM) dendrimer with an ethylenediamine core and amine terminations, to which TPP was added, in HeLa and COS-7 cells.^275^ The transfection efficacy of synthesized G5-TPP dendrimers is much higher than non-modified G5 dendrimers, which might contribute to efficient endosomal escape and mitochondrial targeting ability. HeLa cells (52%) showed a higher transfection efficiency compared to COS-7 cells (40%). This variation might be attributed to differences in endocytic pathways, mitochondrial dynamics, cellular metabolism, and mitochondrial activity between the cancerous (HeLa) and non-cancerous (COS-7) cell lines. Biswas et al. examined a comparable system using a TPP ligand and a G5-PAMAM dendrimer. TPP-modified dendrimers exhibited lower cytotoxicity than TPP-free dendrimers in normal mouse fibroblasts (NIH-3T3). Moreover, the TPP systems exhibited a high degree of selectivity for mitochondria and a strong capacity for cellular internalization.^275,276^

**Inorganic nanoparticles**. Inorganic nanoparticles can facilitate the transmission of large plasmid DNA and small DNA molecules. Rhodamine is a fluorescent chemical that attracts mitochondria, enabling the monitoring of mitochondrial entry into cells and the assessment of mitochondrial membrane potential.^277^ Santos et al. investigated this characteristic of rhodamine for targeting mitochondria. Researchers have created rhodamine nanoparticles using plasmid DNA by utilizing a coprecipitation approach with CaCl₂ and Na_2_CO_3_.^278^ The above inorganic compounds effectively encapsulated several plasmids and promoted cellular endocytosis; their targeting to mitochondria was verified by confocal microscopy experiments.^278^ Costa’s team created CaCO₃–pDNA–Rho123 nanoparticles utilizing the coprecipitation technique.^279^ These vectors formed nanoparticles of an appropriate size and surface charge after encapsulating plasmids with the GFP gene. In fluorescence confocal microscopy images, the nanoparticles showed targeted delivery into the mitochondria of fibroblasts and HeLa cells.^279^ Costa et al. also constructed a compound [16]phenN_2_ fluorescently labeled calcium carbonate delivery system in which *p53* and *ND1–GFP* plasmids were encapsulated to achieve therapeutic effects of targeted intervention in mitochondria.^280^ Confocal fluorescence images showed that [16]phenN_2_ fluorescence intensity was significantly greater in mitochondria than in the cytoplasm or lysosomes.^280^

##### Biological approaches

**Mitochondria-targeting signal peptides**. Given that the precursor proteins of most mitochondrial proteins are synthesized in cytoplasmic ribosomes, these precursor proteins require specific MTSs that can be recognized by mitochondrial surface receptors and transported into the mitochondrion via membrane passage orientations.^281^ The translocase of the outer membrane (TOM) complex is the primary entry point for the recognition of MTS sequences to induce the entry of therapeutic tools into the mitochondria, and the entry channel subunit is an intact membrane β-barrel protein, Tom40. Upon crossing the TOM complex, the protein substrate containing the presequence is delivered to the inner membrane via the translocase of the inner membrane (TIM) complex, and the TOM and TIM form a supercomplex during translocation to achieve effective input of therapeutic tools across the mitochondrial double membrane.^281–286^ Khan et al. designed a protocol delivery system comprising three domains: (i) protein transduction domains that promote cellular uptake, (ii) mitochondrion-targeted MTS domains, and (iii) TFAM that recombines with wild-type mitochondrial DNA.^286,287^ Targeted transport of protein transduction domains (PTD)–MTS–TFAM to mitochondria can effectively reduce the number of mutated mitochondrial DNA copies, improve the cellular mitochondrial respiration rate, and increase the DNA copy number.^288^ Injecting PTD–MTS–TFAM compounded with wild-type mtDNA into mice via the tail vein can effectively increase the respiratory rate of the mouse brain and skeletal muscle mitochondria and simultaneously increase the levels of mtDNA and TFAM, demonstrating a synergistic therapeutic effect.^289–292^ In addition, PTD–MTS–TFAM was also used to construct LHON model by expressing a pathogenic mtDNA carrying the *G11778A* mutation associated with LHON disease.^293^

**Framework of the nucleic acid-based delivery system**. Highly editable DNA has enhanced structural flexibility, allowing lengthy DNA strands to fold into various two- or three-dimensional shapes.^294–296^ The earliest DNA nanostructures were fixed four-arm branches; branch structures and three-dimensional structures, such as DNA tetrahedra, DNA prisms, DNA polyhedra, and DNA nanolanterns, were then developed to meet the need for more complex and diverse DNA nanostructures.^297–299^ More editable and replaceable positions are available given the precise spatial design, flexible programmability, and sequence specificity of DNA nanostructures.^300,301^ Modified DNA framework architectures can transport therapeutic medicines into the mitochondria.^302–304^ Chan et al.^299^ attached MTS to DNA oligomers using a series of chemical modifications, and the modified DNA oligomers were then combined with Cy5-labeled DNA nanocages (DNA-NCs) to form the final MTS DNA-NCs, with additional thymidine units added to prevent steric clashes. The framework structure can be targeted and delivered to mitochondria by connecting the MTS to the branching structure of DNA nanostructures, indicating the huge potential of those nanostructures in targeting organelles.

**Virus-based mitochondrial targeting**. Adenoviruses have been utilized as effective tools for the transportation of mitochondria-targeting nucleases. The mito-ApaLI enzyme has been effectively administered to the hearts of NZB/BALB mice using recombinant adenovirus type 5 to target and remove mitochondrial DNA.^142^ However, the limited duration of expression and robust immunological reactions restrict the use of adenoviruses.^142^

AAV is another commonly utilized viral tool for mitochondrial gene therapy because of its extended expression and established clinical safety. AAV vectors have a maximum packaging capacity of under 5 kilobases. This limitation restricts the transgene size and its effective distribution.^305,306^ Although AAV vectors have limited DNA packaging capacities, they are preferred for in vivo research involving mitochondrion-targeted nucleases. Research has demonstrated that individuals exposed to AAV develop an immunological reaction, which hinders the administration of effective genetic therapy.^306^ Recombinant adeno-associated virus (rAAV) is nonpathogenic and has minimal immunogenicity. Various tissues and cells can be transduced by different AAV serotypes by modifying the tissue specificity of rAAV.^307^ AAV serotypes, such as AAV6 and AAV9, exhibit specificity for skeletal and cardiac muscle. AAVs containing DNA survive as episomes for an extended period in transduced cells, making AAV vectors a potential therapeutic option for homoplasmic or heteroplasmic mutation. These vectors can deliver therapeutic genes to patients’ nerves and muscles.^308,309^ Creating secure and efficient AAV delivery techniques to introduce mtDNA-editing tools into specific cell types is a significant yet worthwhile undertaking.

Recent clinical trials have utilized the nuclear delivery of recombinant AAV to re-express the mtDNA-encoded *ND4* gene, which is mutated in LHON. The efficacy of this impact was demonstrated in a phase 3 clinical study in which visual function was enhanced in individuals diagnosed with LHON.^310–313^ rAAV2, rAAVrh10, and rAAV9 have been utilized in therapeutic settings to transport modified genes to the central nervous system and might be beneficial in treating Alzheimer’s disease (AD) and spinal muscular atrophy.^314^ Various methods, including in silico analysis, rational design, and directed evolution, have been employed to modify AAV capsid proteins, enhancing tissue selectivity and reducing off-target transduction.^315^ Tissue specificity can be attained by utilizing tissue-specific promoters, such as the alpha-1 antitrypsin promoter for liver transduction, PGDF and NSE for neurons, and desmin for skeletal muscle.^316–319^

Additionally, when utilizing AAV viral delivery for mtDNA modification tools, it is important to address the immunogenicity of the AAV capsid, the neutralizing antibodies, and the immunological response to AAV-encoded transgenes. Therefore, creating new AAV serotypes with fewer immune reactions will enable the use of AAV to deliver mtDNA editing tools in clinical therapy. The bacteria-derived toxins FokI, TALE, and DddA may influence T-cell responses.^320^ An altered AAV vector has been created by combining an outer capsid protein with an MTS to deliver DNA to mitochondria in living organisms. It is uncertain how MTS–AAV reaches the mitochondria and releases encapsulated DNA inside the organelle despite the documented effective delivery, recombination, and expression of DNA in the mitochondrial matrix.^321^ Second, there is inefficient recombination in mammalian mitochondrial DNA. Therefore, unlike in yeast or green algae, DNA transferred to the mitochondria cannot be readily incorporated into the host genome. Moreover, due to the polycopy nature of mtDNA, it is challenging to isolate specific mutant mtDNA from other mtDNA combinations to reach the necessary quantities for experimental or therapeutic purposes. Hence, a technique that can decrease the concentration of mitochondrial nuclease-encoding AAV is essential for therapeutic use. Possible solutions include enhancing AAV capsid specificity, implementing temporary immunosuppressive procedures, delivering injections locally to certain organs, and incorporating mitoZFNs or mitoTALEN monomers into a single AAV capsid. The development of AAV technology that can safely deliver mitoZFNs or mitoTALENs while being compatible with the current recombinant AAV manufacturing capacity and clinical standards is a significant challenge.^252,322^ In addition, the significant expense associated with clinical studies and therapies poses a major barrier to implementing mitochondrial gene therapy.^323^

The interplay among physical, chemical, and biological approaches lies in their complementary strengths and limitations. Physical methods provide direct delivery but with low specificity; chemical methods enhance targeting but may involve complex synthesis; biological methods offer high specificity but can be limited by immune responses. Combining elements from each—such as using chemical carriers with biological targeting signals—could lead to more effective mitochondrial gene therapies by maximizing delivery efficiency while minimizing adverse effects.

While mitochondrial gene editing focuses on directly modifying genetic defects, an alternative approach, artificial mitochondrial transfer, offers a different strategy to address mitochondrial dysfunction. This method involves the replacement of damaged mitochondria with healthy ones from a donor, providing a holistic solution by entirely substituting the malfunctioning organelles. Both strategies aim to rectify mitochondrial deficiencies but differ fundamentally in their execution and potential applications.

### Artificial mitochondrial transfer

#### Mitochondrial transfer observed in health and disease

The initial observation of organelle migration across mammalian cells through tunneling nanotubes (TNTs) was made by Rustom et al. in 2004,^74^ and the initial demonstration of functional mitochondrial transfer from mesenchymal stem cells (MSCs) to cells with dysfunctional mitochondria was conducted by Spees et al. in 2006.^75^ Subsequently, mitochondrial transfer was observed between different cells, tissue types, and even species. Extracellular vesicles (EVs)-containing mitochondria and free mitochondria were later observed during the transfer process.^76^ The transferred mitochondria may have significant and enduring consequences due to the potential for cross-species fusion between foreign mitochondria and host cell mitochondria.^324^ In vitro, mitochondria function for a minimum of 45 cell passages, equivalent to 135 days.^325^

Mitochondrial transfer contributes to the structural and functional connectivity of tissue homeostasis and is related to recipient-cell mitochondrial supplementation, donor-cell mitochondrial quality control, and cell-to-cell signal transduction. The detailed molecular mechanism of mitochondrial transfer has been reviewed in previous reviews.^326,327^

##### Transfer between differentiated cells

Mitochondrial transfer is mainly observed in vitro in cultures of the same cell type (for example, mitochondria between normal rat kidney cells by TNT^328^) or in cocultures of different types of cells (for example, mitochondria exchange between cardiomyocytes and cardiofibroblasts,^329^ between rat cardiomyocytes and human endothelial progenitor cells,^330^ and between mouse osteocytes and endothelial cells (ECs) of transcortical vessels^331^).

In recent years, evidence of mitochondrial transfer in the neural systems has been reported, and a primary network has been constructed that might provide some guidance for studies in other systems. In the nervous system, mitochondrial transfer is multidirectional. Neurons can be the donor, transferring mitochondria to neurons and glial cells: functional mitochondria can be transferred from normal neuroblastoma cells to mitochondria-defective neuroblastoma cells through tunnel nanotubes,^332^ and neurons at the optic disc release a significant quantity of mitochondria, which are then broken down by the lysosomes of nearby glial cells.^333^ It was assumed that the transcellular degradation of mitochondria occurs in other regions of the central nervous system due to structurally comparable accumulations of deteriorating mitochondria along neurites in the superficial regions of the cortex. Conversely, neurons can be recipients, internalizing mitochondria from neurons (stated above), astrocytes, and macrophages. Astrocyte-derived mitochondria normalize the neuronal calcium dynamics impaired by cisplatin,^334^ protecting neurons against apoptosis in ischemia.^335^ Macrophages play an active role in the remote modulation of inflammatory pain elimination by delivering mitochondria to sensory neurons.^336^ Additionally, mitochondria secreted by astrocytes can enter microglia, improving recovery from intracerebral hemorrhage.^337^ Mitochondrial transfer can occur in multiple stages in the nervous system: dysfunctional mitochondria spread from microglia to astrocytes to neurons.^338^

##### Transfer related to stem cells

Stem cells possess substantial differentiation potential and high levels of mitochondrial activity. As an optimal mitochondrial resource, stem cells provide functional mitochondria and facilitate the homeostasis of target cells. In an in vitro ischemia-reperfusion model, the restoration of injured endothelial cells was achieved through the transfer of mitochondria via TNTs between MSCs and human umbilical vein endothelial cells (HUVECs).^339^ Bronchial epithelial cells obtain functional mitochondria when cocultured with mesenchymal stromal cells, regardless of whether the bronchial epithelial cells are derived from the lung parenchyma or bronchoalveolar lavage fluid.^340^ Interestingly, in addition to restoring physiological functions, transferred mitochondria appear to have the potential to reprogram differentiated cells. The reprogramming of cardiomyocytes to a more immature state was achieved through the transfer of mitochondria from human multipotent adipose-derived stem cells to cardiomyocytes.^341^ Mitochondrial transfer from stem cells appears to provide somatic cells with differentiation abilities to a certain extent. Conversely, somatic cell-derived mitochondria provide differentiation cues to stem cells. The regulation of MSC differentiation in coculture necessitates the transfer of mitochondria from vascular smooth muscle cells (VSMCs) to MSCs.^342^ Similarly, the bidirectional mitochondrial transfer between human mesenchymal multipotent stromal cells (MMSCs) and rat RTCs induces the differentiation of MMSCs into kidney tubular cells.^343^ More stem cell artificial mitochondrial transfer therapies are discussed in detail in the preclinical research section.

##### Transfer related to cancers

Mitochondria play a vital role in most metabolic pathways, as they contribute significantly to the synthesis of energy and biomass while also serving as metabolic sensors. From this perspective, mitochondria finely regulate signaling pathways linked to cancer cell metabolism.^344^

**From host cells to cancer cells**. The equilibrium between glycolysis and OXPHOS in cancer cells can be modified to align with biomass generation and energy requirements. Regardless of whether cancer cells use OXPHOS or glycolysis as their main method of energy generation, pathways with excessive rates contribute to detrimental effects on mitochondria. Increasing evidence reveals that mitochondrial transfer from host cells helps cancer cells overcome mitochondrial impairment.

Wharton’s jelly-derived MSCs transfer their mitochondria to mtDNA-depleted osteosarcoma cells (143B) and restore the original defective OXPHOS.^325^ Similarly, human MSCs deliver mitochondria to 143B cells lacking mitochondrial function to rescue proliferation.^345^ Host mtDNA can also be transferred to tumor cells with impaired respiratory function, restoring respiration and promoting tumor initiation.^346^

**Transfer between cancer cells**. Cellular interactions in the tumor microenvironment have been extensively studied in tumor development.^347^ Mitochondrial transfer between cancer cells has been found to contribute to the tumor communication network. Mitochondria are transmitted in a bidirectional manner between cancer cells, such as primary cells from human laryngeal squamous cell carcinoma,^348^ mesothelioma cells (MSTO-211H),^349^ and GBM stem-like cells.^350^ In addition to the simple exchange of mitochondria between cancer cells of the same type, alterations in the characteristics of cancer cells after receiving mitochondria from the microenvironment have been observed. Cancer-associated fibroblasts facilitate the aggressiveness of prostate cancer (PC3, DU145) through mitochondrial transfer.^351^ Mitochondrial transport from aggressive bladder cancer cells (T24) to nonaggressive bladder cancer cells (RT4) has been found to increase bladder cancer cell invasiveness.^352^ Coinjection of human cancer-associated fibroblasts (CAFs) and human prostate cancer (PCa) cells into severe combined immunodeficient mice showed that mitochondria from CAFs enhance the motility and lactate metabolism of cancer cells.^351^

**Mitochondrial transfer and chemoresistance**. Regardless of chemotherapy or radiotherapy, anticancer medicines result in mitochondrial impairment via oxidative or related stress, and chemoresistance mediated by mitochondrial transfer is a major challenge: both the transfer of mitochondria into cancer cells (normal mitochondrial replenishment) and the transfer of mitochondria from cancer cells (abnormal mitochondrial clearance) can result in chemoresistance. Acute myeloid leukemia (AML)-derived cells acquire normal mitochondria from cocultured murine or human bone marrow stem cells (BMSCs), leading to increased survival rates after chemotherapy.^353^ MSCs also transport mitochondria to rescue acute lymphoblastic leukemia (ALL) B-cell precursors from ROS-inducing chemotherapy.^354^ Tumor-activated stromal cells increase GBM proliferation and resistance to standard treatments via mitochondrial transfer.^355^ In the tri-culture system (MCF7 cells, MSCs, and E4+ECs), mitochondrial transfer exhibited comparable patterns between cancer cells and MSCs or ECs. However, the exchange of mitochondria between ECs and cancer cells is more pronounced, increasing cancer cell chemoresistance.^356^ Pheochromocytoma (PC) 12 cells can survive after ultraviolet light (UV) treatment by obtaining functional mitochondria from untreated PC12 cells.^357^ Mitochondrial transfer from Jurkat cells to MSCs may also result in chemoresistance since damaged mitochondria are removed from cancer cells.^358^

However, the detailed mechanisms of chemoresistance via mitochondrial transfer remain unclear. Conflicting reports exist regarding the effect of mitochondrial transfer on cancer cells. The proliferation of cancer cells (MCF-7/ADR) was found to be inhibited by normal mitochondria derived from epithelial MCF-12A cells, whereas susceptibility to chemotherapeutic drugs was enhanced.^359^ The authors stated that “ROS will be elevated” and that “depriving cancer cells of glycolytic intermediates”^360^ could explain their results. However, these findings might be oversimplified since the effects of new mitochondria are multifaceted, especially in different cancer types. For example, mitochondrial transfer has been especially studied in AML since ATP generation in AML depends on oxidative phosphorylation, contrary to the common Warburg effect in solid tumors.^361^ In vivo, normal mitochondria are transferred from human BMSCs to leukemic blasts, causing multiple myeloma cells to rely on oxidative phosphorylation.^362,363^ Future investigations might provide insight into the unique role of energy metabolism in AML and novel therapeutic strategies.

#### Technologies for artificial mitochondrial transfer

##### Mitochondrial imaging technologies

Fluorescent dyes are widely employed as a prevalent technique for quantifying mitochondrial transport in vitro.^364^ The use of fluorescent dyes such as mitoTracker provides a visible and quantitative method for studying mitochondrial transport in vitro. Fluorescent dyes enable the continuous tracking of mitochondrial transport in real-time via flow cytometry or confocal microscopy.^365,366^ mtDNA sequencing is an alternative technique employed to quantify mitochondrial transport in vitro via the identification of donor mtDNA in recipient cells.^367^ The mtDNA sequencing technique can even identify specific mitochondria with edited genes. Furthermore, Zhang et al. utilized single-cell sequencing techniques to investigate the dynamics of mitochondrial transport between cancer cells and T cells via a novel deconvolution process.^368^ Applying this method, they accurately predicted the recipient cells and their relative mitochondrial compositions and identified a reproducible mitochondrial transfer phenotype.

Fluorescent mitochondrial labeling and mtDNA sequencing^369^ can also be performed in vivo. Special mtDNA sequencing focused on mtDNA haplotypes has also been frequently used in vivo. Mitochondrial DNA haplotypes are specific sequences of mitochondrial DNA that can aggregate with the mtDNA of other mitochondria, providing insights into evolutionary origins. MtDNA haplotypes are highly valuable for investigating mitochondrial trafficking from mothers to fetuses during pregnancy.^370^

Advanced imaging techniques have facilitated the observation of mitochondrial transport within living organisms. One such technique involves using two-photon microscopy to monitor the movement of mitochondria in vivo.^371^ This approach enables the real-time imaging of mitochondrial dynamics and intercellular travel, providing essential insights into the fundamental mechanics of mitochondrial trafficking in vivo.

##### Mitochondrial transfer technologies

Currently, the mitochondrion is acknowledged as an endosymbiotic entity whose noneukaryotic derivation might increase its capacity for intercellular transplantation and fusion with the recipient cells’ original mitochondrial network.

Coculture was the first and most widely used artificial mitochondrial transfer method^72^ and has been demonstrated to be effective directly and in transwell systems.^372^ The coculture technique has notable benefits regarding safety and simplicity; nonetheless, it is essential to acknowledge that heterogeneity and efficiency pose considerable limitations. Therefore, many new methods have been developed to achieve reliable and efficient artificial mitochondrial transfer. Notably, there are several difficulties associated with artificial mitochondrial transfer. First, how to precisely drive mitochondria to move toward target cells is an issue. Second, naked mitochondria might be toxic to cells when located outside the cell membrane. An illustration of this phenomenon is the activation of proinflammatory processes in leukocytes by oxidized cardiolipin present on the mitochondrial membrane.^373^ Third, the mechanisms regulating the fate of transplanted mitochondria have not been fully elucidated.

The exploration of artificial mitochondrial transfer can be classified into two categories. The first requires the use of additional physical force to facilitate the internalization of free mitochondria into recipient cells, including microinjection,^374,375^ magnetomitotransfer,^376^ mitoCeption,^377,378^ photothermal nanoblade,^379^ mitoPunch,^380,381^ optical tweezers,^382^, and droplet microfluidics.^383^ The second adopts biological modifications or vectors to facilitate delivery, including small molecule peptides,^384^ polymers,^385^ and lipids,^386^ to optimize mitochondrial internalization.

**Fig. 5:**
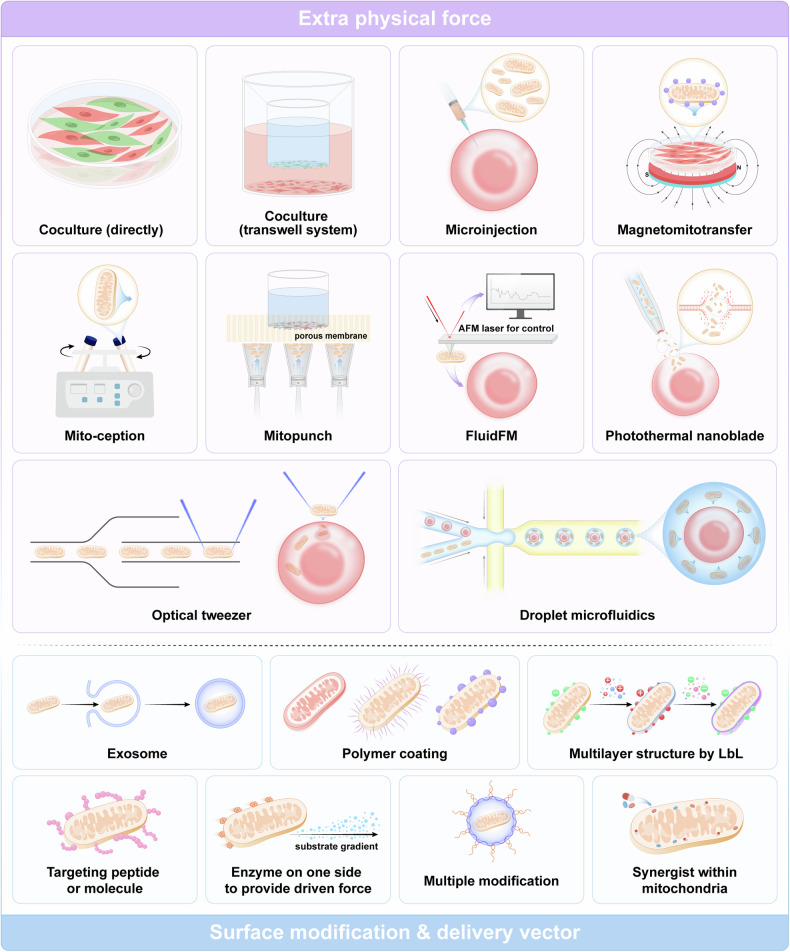
Strategies for artificial mitochondrial transfer. Physical methods for facilitating the transplantation of free mitochondria into cells include coculture, microinjection, magnetomitotransfer, mito-ception, mitopunch, fluidFM, photothermal nanoblade, optical tweezers, droplet microfluidics, etc. These methods utilize natural intercellular mitochondrial transfer or apply additional driving forces using various devices. Biochemical methods involve utilizing mitochondrial surface modifications or vectors for delivering mitochondria, including exosomes, polymer coatings, targeting peptides, and so on. (Generated by the authors with Adobe Illustrator)

**Extra physical force**. The advantage of using additional physical force is that the manipulator can determine the appropriate timing of the transplant and the number of transplanted mitochondria. However, these approaches are device-dependent and challenging to use in vivo (Fig. 5).

Microinjection of exogenous mitochondria was initially applied in oocytes harboring mutants related to mitochondrial disease.^374,375^ Microinjection is appropriate for manipulation and achieving exact efficacy for a single germ cell. Procedures with greater efficiency and larger scale are needed for diseases related to the degeneration of mitochondria in multiple cells. Magnetomitotransfer adds magnetic bead-labeled mitochondria to recipient cells under the magnetic field produced by a specialized device.^376^ mitoCeption^377,378^ involves using centripetal force (~1500 g) created within a centrifuge to localize mitochondria to target cells. The mitoPunch platform is a highly parallel, pressure-driven instrument designed for large-scale transplantation.^380,381^ The mitoPunch technique employs a plunger to induce the physical transformation of a flexible reservoir that houses isolated mitochondria. The plunger’s motion propels the mitochondria in the reservoir through a membrane with tiny holes, where adherent cells are cultivated.^387^ In contrast to mitoCeption, this rapid, cost-effective, and high-throughput method can be used in any laboratory setting. Additionally, this method requires a minimal quantity of donor mitochondria.^380^ Both mitoPunch and mitoCeption can generate cells that permanently retain exogenous mtDNA.^380^

FluidFM^388,389^ uses hollow cantilevers for artificial mitochondrial transfer with atomic force microscopy (AFM)-assisted control. Researchers have integrated AFM and nanofluidic techniques to achieve force and volume control under real-time inspection. The photothermal nanoblade^379,390^ has a modified microneedle using a laser-induced bubble to generate perforations in the cell membrane, facilitating the efficient transport of mitochondria into the cell via coordinated flow. Optical tweezers employ a concentrated light beam to generate trapping force magnitudes in the range of piconewtons.^391^ Shakoor et al. used an automated optical tweezer-based micromanipulation system to control transplantation.^382^ This device combines optical tweezers and automated systems to transport healthy mitochondria to recipient cells using endocytosis with high accuracy and efficiency. Droplet microfluidics selectively distributes a continuous stream of items to be processed into distinct droplets of micrometer dimensions.^383^ The enclosed microenvironment of the droplets decreases the distance that the isolated mitochondria have to travel and increases their chances of coming into contact with the cell, improving the effectiveness of artificial mitochondrial transfer. The quantitative control of mitochondria transplantation can be achieved by altering the proportion of the mitochondrial solution.^383^

**Surface modification and delivery vectors**. Although cell-free mitochondria are reportedly abundant, normal components of blood,^76,392^ surface modification, and delivery vectors have several benefits in the application of artificial mitochondrial transfer.

1. Enhanced stability and internalization.
  Although spontaneous mitochondrial transport has been widely detected in coculture systems, two unexpected outcomes of transplanted mitochondria in recipient cells have been documented: 1. The absence of modifications in mitochondria may result in their failure to escape endosomes, subsequently leading to their degradation. 2. During spontaneous transfer, mitochondria undergo degradation after their internalization.^333,335^ Therefore, one main goal of mitochondrial modification and delivery is to protect mitochondria (prolonging circulation durations by preventing protein adsorption and identification by macrophages, a phenomenon commonly referred to as “stealthiness”),^393^ enhance stability, improve internalization, and avoid direct exposure to the membrane.
  Extracellular vesicles are potential candidates since mitochondria can be naturally secreted via EVs.^394,395^ With a mitochondria-specific dye or size filter, EVs containing mitochondria can be easily screened out. However, one main limitation of this strategy is that the mitochondria naturally secreted via EVs are often damaged,^396,397^ limiting their therapeutic effects.
  The outer membranes of mitochondria are abundantly enriched with anionic lipids that are negatively charged under biological circumstances.^398,399^ Due to this characteristic, charge-based mitochondrial coatings have been researched and demonstrated to be reliable and effective. Cationic gelatin nanospheres have the potential to be readily immobilized on the mitochondrial surface through electrostatic interactions, enhancing the efficiency of mitochondrial internalization.^400^ Comparably, coating with a mixture of DOTAP and DOPE using a reverse emulsion technique^386^ or with the dextran‐TPP polymer^385,401^ also improved artificial mitochondrial transfer and efficacy. Furthermore, a layered architecture with flexible thickness could be created using the layer-by-layer (LbL) approach. LbL is an effective option for modifying mitochondria with minimal disruption of their natural bioactivity.^402^ Chen et al. identified chitosan and poly(acrylic acid) (PAA) as promising options for mitochondrial LbL modification owing to their exceptional compliance with mitochondrial membranes.^403^ The researchers detected the presence of a nanomaterial coating with a thickness of ~45 nm after six rounds of LbL assembly. This coating effectively preserved the integrity of the mitochondrial membrane, resulting in no noticeable distortion or collapse. Interestingly, charge-based coatings also overcome the barrier of electrostatic repulsion when the target cells, for example, cancer cells, are negatively charged.^403^
2. Enhancing the ability to penetrate
  Maeda et al. reported that cell penetration peptide (CPP) and glucan modification of mitochondria can significantly increase the efficiency of artificial mitochondrial transfer, reducing oxidative stress and apoptosis in cardiomyocytes.^404^ Similar improvements in penetration were widely reported when CPP-modified mitochondria were applied to treat mitochondrial disease,^384^ breast cancer cells (MCF-7 cells),^405^ and dopaminergic neurons.^406^
  Engineered mitochondria incorporating a Janus surface structure were also developed, drawing inspiration from the natural bacterial mechanism. This system utilizes glucose oxidase to drive movement, enabling it to navigate toward tumors and autonomously infiltrate the depths of tumor tissues. Furthermore, the system exhibited a prolonged retention period.^407^
3. Incorporate additional synergists
  A two-in-one strategy involving mixing mitochondria in EVs and exogenous 27-kDa heat shock protein (HSP27) has been developed to preserve brain endothelial cell tight junction integrity.^394^ A synthetic biological system has been created to enhance photodynamic therapy by combining a photosensitizer with mitochondria.^408^ In addition to the direct combination of mitochondria and synergists, optogenetic tools have been applied to modify the mitochondrial membrane to realize light-activated engineering.^409^

##### Artificial mitochondria

In several recent studies, researchers have attempted to construct artificial mitochondria by transplanting existing ATP-producing structures. *Escherichia coli* cells were successfully transplanted into bacteriogenic protocells to allow mitochondria to produce ATP.^410^ Another study revealed that camouflaged nanophotosynthetic thylakoids could achieve quick penetration into chondrocytes by fusing with the cell membrane, preventing the destruction of the lysosomes.^165^ Moreover, exposure to light increases intracellular ATP.^165^ Although these novel strategies are promising, more researchers have adopted a from-bottom-to-up method. According to this theory, artificial mitochondria have three fundamental components: the membrane, the proton gradient generator, and the ATPase.^411^ The membranous compartment separates the reactive site from the outer environment. The generator produces a proton gradient through the membrane, which the ATPase subsequently utilizes to facilitate the synthesis of ATP.

**The membranous compartment**. Lipids were most frequently used in the initial attempts to form the membranous compartment, whereas novel strategies adopting synthetic polymers were also appealing because of their stability, flexibility, and durability.^412^ Specific synthetic polymers automatically form stable vesicles^413^ with dimensions similar to those of a typical lipid bilayer (5 nm).^414^ Moreover, combining synthetic polymers with lipids makes it possible to create hybrid lipid‒polymer membranes exhibiting tunable distribution. Hybrid membranes have demonstrated substantial potential in artificial mitochondria.^415,416^

Researchers have attempted to integrate multiple optimally reconstituted elements into one compartment using biological-mediated^417^ or charge-mediated^418^ methods. Biologically mediated methods reconstitute each protein separately in liposomes and then fuse the different membrane protein-containing vesicles to construct a vesicle containing multiple membrane proteins without altering the orientation of any component.^419,420^ Charge-mediated fusion, the fusion of liposomes containing cationic lipids with liposomes with a negative charge, was first described in 1988.^421^ Charge-mediated fusion has a significantly shorter duration (for several minutes) than biologically mediated fusion but preserves the orientation of the membrane protein.^418^

**The proton gradient generator**. The fundamental approach employed in artificial mitochondrial models to generate transmembrane proton gradients involves the utilization of oxidation or electron transport of specific substrates catalyzed by a sequence of enzymes.

Most ATP in living cells is generated through the collaborative activity of respiratory complexes within the mitochondria. However, integrating all the complexes into a single compartment in artificial structures is challenging because each protein needs an individual specialized mechanism for reconstitution.^422^ Hence, employing a solitary constituent derived from the electron transport chain is a pragmatic approach.^423,424^ Similarly, artificial bacterial respiratory system components are effective; for example, cytochrome bo3.^418,425–427^ In addition, glucose oxidase can generate protons via oxidative reactions.^428–430^

Self-adaptation or homeostasis maintenance, when the external environment is altered, is impossible for the above artificial mitochondria, and the efficiency of ATP generation may correspondingly decrease significantly. In particular, fluctuations in osmotic pressure can impact synthetic mitochondrion behavior and perhaps lead to the destruction of these synthetic organelles. Further investigations are needed to determine the ability of synthetic mitochondria to generate ATP sustainably across diverse situations. More information on the process of artificial mitochondria synthesis could be found in Biner et al.’s review.^422^

## Application of engineered mitochondria in disease

**Fig. 6:**
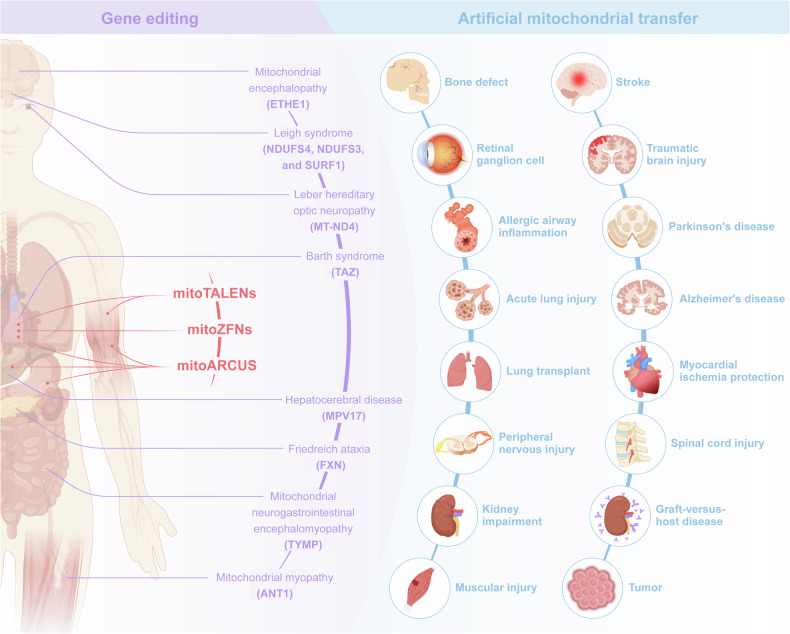
Preclinical research on the application of engineered mitochondria in diseases. Only limited in vivo studies have demonstrated the effectiveness of mitoTALEN, mtZFN, and mitoARCUS in clearing mutant mtDNA in specific tissues (in red). Concurrently, various mitochondrial diseases involving different mutant genes have been extensively investigated in vivo to determine the therapeutic effects of nuclear genome editing (in purple). With the rapid advancement in mitochondrial gene editing, the application may be soon extended, particularly to scenarios that have been researched for previous nuclear genome editing. The administration of artificial mitochondrial transfer in multiple animal models with diseases related to mitochondrial dysfunction has shown promising results, including in the cardiovascular, nervous, digestive, visual, urinary, and musculoskeletal systems (in blue) (Generated by the authors with Adobe Illustrator)

The field of engineered mitochondria has seen significant strides through the development of both mitochondrial gene editing and artificial mitochondrial transfer. Mitochondrial gene editing enables precise alterations to mitochondrial DNA, offering a targeted approach to rectifying genetic defects at their source. Meanwhile, artificial mitochondrial transfer provides a method to replace dysfunctional mitochondria with healthy ones, thus restoring cellular function. These complementary approaches not only highlight the potential for innovative treatments but also underscore the versatility of engineered mitochondria-based therapies. By now, an increasing number of preclinical and clinical studies have yielded encouraging results (Fig. 6).

### Application of gene-edited mitochondria in disease treatment

Gene editing strategies targeting mitochondrial diseases encompass two distinct approaches: the editing of nuclear genes encoding mitochondrial-related proteins and the direct editing of mitochondrial genes. Both approaches have shown promising progress in preclinical studies (Table 3).

**Table 3:** Preclinical studies on mitochondrial gene editing

Author	Diseases	Therapeutics	Delivery method	Animal model	Main result
Nervous system					
Di, 2012^435^	Ethylmalonic acid encephalopathy	*hETHE1* cDNA	ssAAV2/8 vector	*Eth1* KO mice	Improvement of the disease’s primary metabolic and biochemical indicators
Reynaud-Dulaurier, 2020^437^	Leigh syndrome	*Ndufs4*-IRES	AAV9	*Ndufs4* KO mice	Reduced gliosis, significant body weight and locomotor activity improvement, and a significantly prolonged survival time
Digestive system					
Marta, 2021^438^	Mitochondrial neurogastrointestinal encephalomopathy	CRISPR/Cas9 and *TYMP* cDNA	Lipid nanoparticle or polymeric nanoparticle	*Tymp/Upp1* mice	Promoted the synthesis and secretion of *TYMP* mRNA and active enzymes in the liver
Battani, 2014^439^	Mitochondrial hepatocerebral disease	*MPV17*	AAV8	*MPV17* KO mice	The supramolecular complex level of *MPV17* was effectively restored, and the progression of liver failure was delayed
Musculoskeletal system					
Flierl, 2005^441^	Mitochondrial myopathy	*ANT1* cDNA	AAV2	*ANT1* KO mice	Improved mitochondrial functions and reversed histopathological and clinical symptom changes
Perdomini, 2014^473^	Friedreich’s ataxia	*hFXN* cDNA	AAVrh10	*Mck-Cre-Fxn*^*L3/L–*^ mice	Improved frataxin protein expression, delayed the progression of myocardial fibrosis, and prolonged the survival time
Myocardial disease					
Bacman, 2018^115^	Mitochondrial cardiomyopathy	mitoTALENs	AAV9	*m.50624* *C* > *T* mice	Restored the normal expression of tRNA^Ala,^ and ameliorated the symptoms of mitochondrial cardiomyopathy.
Gammage, 2018^146^	Mitochondrial cardiomyopathy	mitoZFNs	AAV9.45	*m.50624* *C* > *T* mice	Restored mitochondrial tRNA^Ala^ expression levels in the heart and improved tissue levels of lactate, pyruvate, and aspartate
Zekonyte, 2021^70^	Mitochondrial cardiomyopathy	mitoARCUS	AAV9	*m.50624* *C* > *T* mice	Reduced the amount of mutant mtDNA in organs by inducing specific dsDNA breaks

*AAV* adeno-associated viruses, *ANT1* adenine nucleotide translocator 1, *Cas9* CRISPR-associated protein 9, *CRISPR* clustered regularly interspaced short palindromic repeats, *hETHE1* human ethylmalonic encephalopathy protein 1, *hFXN* human frataxin, IRES intra-ribosome-entry-site, *KO* knockout, *mitoARCUS* mitochondrial-targeted meganuclease, *mitoTALENs* mitochondrial-targeted transcription activator-like effector nucleases, *mitoZFNs* mitochondrial-targeted zinc finger nucleases, *Ndufs4* ubiquinone oxidoreductase subunit S4, *TYMP* thymidine phosphorylase, *Upp1* uridine phosphorylase 1

#### Nuclear genome editing in mitochondrial diseases

Ethylmalonic acid encephalopathy (EE) is a type of mitochondrial encephalopathy resulting from toxic sulfide buildup due to mutations in the *ETHE1* gene. The *ETHE1* protein encoded by the *ETHE1* gene is a mitochondrial sulfur dioxygenase located in the mitochondrial matrix that is involved in sulfur metabolism and converts sulfides into less toxic substances. *ETHE1* protein deficiency leads to the accumulation of hydrogen sulfide (H_2_S) and ethylmalonic acid (EMA) in the brain, decreased enzyme activity, and mitochondrial energy metabolism disorders.^431^ H2S is toxic at relatively high concentrations and inhibits enzymes such as cytochrome c oxidase (COX)^432^ and short-chain acyl-CoA dehydrogenase,^433^ resulting in intellectual and motor lag, persistent diarrhea, skin ecchymosis and petechiae, orthostatic cyanosis of hands and feet, and brain gray matter damage.^434^ Common brain MRI abnormalities include symmetrical basal ganglia and brainstem necrotic lesions, similar to mitochondrial encephalopathy. Half of affected children die in the first 2 years due to metabolic crisis, and there is currently no specific treatment for this disease. Di et al. injected a ssAAV2/8 vector carrying human *hETHE1* cDNA into *Eth1* KO mice, significantly improving symptom phenotypes and survival during the early stages of EE disease. This impressive outcome was linked to the improvement of the disease’s primary metabolic and biochemical indicators, such as EMA and thiosulfate levels in plasma and COX activity in tissues.^435^ Leigh syndrome is a progressive encephalopathy caused by defects in the *NDUFS4, NDUFS3*, and *SurF1* genes and is the most common childhood mitochondrial disease.^436^ Leigh syndrome can result in a wide range of neurodegenerative disease phenotypes, including ataxia, dystonia, vision loss, and hearing loss. Reynaud-Dulaurier et al. intravenously injected AAV-PHP into *Ndufs4*-KO adult mice and showed reduced gliosis, significant body weight and locomotor activity improvement, and a significantly prolonged survival time.^437^

Mitochondrial neurogastrointestinal encephalomopathy is a rare multisystem disorder caused by mutations in the *TYMP* gene encoding thymidine phosphorylase (TP) and is inherited as an autosomal recessive disorder. The *TYMP* gene encodes thymidine phosphorylase, an enzyme necessary for the breakdown and transformation of certain nucleosides in the body. Mutations in the TP gene result in very low thymidine phosphorylase activity in cells and tissues. A lack of TP can lead to abnormally high levels of nucleoside, deoxyuridine, and thymidine in the body. Cabrera and her colleagues applied liver-specific promoter sequences, including TBG, PGK, HLP, and AAT, to drive *hTYMP* expression in a deoxyribonucleoside stress model. Compared with the activity of the commonly used promoter PGK, the activity of the liver-specific promoter sequence AAV2/8 is high in *TYMP* transgenic mice. Even at the lowest intravenous injection dose of 5 × 10^11^, the liver TP activity of knockout mice aged 8–11 weeks was effectively restored, and dThd levels returned to near-normal levels.^316^ Marta et al. utilized lipid nanoparticles or polymeric nanoparticles to synergistically deliver CRISPR/Cas9 and *TYMP* cDNA to liver cells, achieving targeted integration of the *TYMP* gene into the liver cells of the *Tymp/Upp1* mouse model. The experimental results showed that after treatment, *Tymp/Upp1* promoted the synthesis and secretion of *TYMP* mRNA and active enzymes in the liver in *Tymp/Upp1* mice, resulting in an effective decrease in plasma nucleoside levels that persisted for 1 year.^438^ Similarly, Battani et al. investigated mitochondrial hepatocerebral disease and delivered the *MPV17* into an *MPV17* knockout mouse model gene using AAV8; the supramolecular complex level of *MPV17* was effectively restored, and the progression of liver failure was delayed.^439^

The adenine nucleotide transporter protein encoded by *ANT1* is the most abundant protein in the mitochondrial inner membrane. Under normal circumstances, the biological function of *ANT1* is to form nonspecific pores that cause mitochondrial inner membrane permeability, output ATP to exchange for cytoplasmic ADP, and act as an electric pump.^440^ *ANT1* mutations cause mitochondrial myopathy (MM), characterized by ragged red muscular fibers and progressive external ophthalmoplegia due to extraocular muscle paralysis. Flierl et al. were the first to conduct preclinical gene therapy research on MM by constructing an AAV2 vector carrying *ANT1* cDNA and injecting it intramuscularly into *ANT1* knockout mice. The *ANT1* knockout mice exhibited significant exercise intolerance and clinical manifestations of metabolic acidosis. After AAV2 vector injection of *ANT1* cDNA, *ANT*1 was stably expressed in muscle precursor cells and differentiated muscle fibers long-term, effectively improving mitochondrial respiratory efficiency and energy metabolism and reversing MM-related histopathological and clinical symptom changes.^441^ Wang et al. used intravenous *TAZ* gene replacement by AAV in a cardiomyocyte-specific *TAZ* knockout mouse model of Barth syndrome; *TAZ* gene replacement effectively prevented the progression of cardiomyopathy and fibrosis in a dose-dependent manner.^442^ Friedreich’s ataxia (FRDA) is caused by mutations in the *FXN* gene that result in severely reduced levels of expression of its encoded protein frataxin, leading to a reduction in iron-sulfur clusters and ATP production, which in turn affects neurological, cardiac, and pancreatic functioning in patients, with approximately 60% of patients passing away from cardiovascular problems.^443^ Perdomini et al. injected AAVrh10-CAG-*hFXN* into Mck-CKO mice, which effectively improved frataxin protein expression, delayed the progression of myocardial fibrosis, and prolonged the survival time.^444^

Over the past 10 years, clinical trials on gene therapy for mitochondrial diseases have mostly concentrated on the most common mutation in LHON: the *m.11778G* > *A* variation in *MT-ND4*.^445^ GS010, a recombinant AAV vector serotype 2 containing cDNA for the wild-type *ND4* protein, was unilaterally injected intravitreally in clinical studies (NCT02652780 and NCT02652767). This treatment led to visual improvement in persons with LHON.^323,446,447^ Studies conducted in retinal ganglion cells and patient fibroblasts showed that the normal *ND4* generated by the therapeutic gene appropriately moved to mitochondria due to MTS.^448,449^ On the ETDRS visual acuity chart, the best corrected visual acuity (BCVA) of the treated eyes improved by an average of 15 letters, while that of the eyes treated with sham surgery improved by an average of 13 letters.^323^ The visual improvement in untreated eyes may be due to the diffusion of rAAV2/2-*ND4* between the optic nerve and the chiasma.^323,446,450^ Phase III studies have demonstrated that intravitreal administration of rAAV2-*ND4* leads to enhanced visual acuity, exhibits favorable safety and systemic tolerance, and significantly advances the regulatory approval procedure.

#### Mitochondrial genome editing in mitochondrial diseases

Programmable nuclease-based mitochondrial gene editing tools are gradually being applied in preclinical studies. Bacman *et al*. intramuscularly injected mitoTALENs into the *m.50624C* > *T* mouse model of mitochondrial cardiomyopathy at a 1.0–1.5 × 10^12^ vg/dose of AAV9 to specifically recognize the disease-causing variant; this approach induced the degradation of the variant in muscle and heart via mtDNA cleavage, effectively restored the mtDNA level to that of the WT, restored the normal expression of tRNA^Ala^, and ameliorated the symptoms of mitochondrial cardiomyopathy in mice.^115^ In a similar *m.50624C* > *T* mouse model, Gammage et al. injected the mitoZFNs-containing AAV 9.45 vector intravenously at a dose of 5 × 10^12^ vg, and their experimental results showed that mitochondrial tRNA^Ala^ expression levels in the heart of the mice were also effectively restored and that the tissue levels of lactate, pyruvate, and aspartate were improved.^146^ Zekonyte *et al*. delivered mitoARCUS into *m.50624C* > *T* juvenile and adult mouse models using an AAV9 vector, which effectively reduced the amount of mutant mtDNA in organs such as the heart, skeletal muscles, kidney, and liver by inducing specific dsDNA breaks; additionally, the level of mt-tRNA^Ala^ expression was significantly increased.^70^

As recently emerging technologies, mitochondria base editors have not been directly used in disease therapy in vivo; however, some initial studies have demonstrated the substantial potential of these tools in this setting. DdCBEs successfully edit mtDNA in the cardiac tissue of mice after delivery by AAV in vivo.^163^ Similarly, zinc finger-DdCBEs delivered by AAV, which are capable of base editing of both mitochondrial and nuclear DNA in vivo, have also been developed.^173^ In addition, the off-target effect of zinc finger DdCBEs is reduced by impeding spontaneous split DddA reassembly.^173^

In addition, mtDNA base editing technology can induce the generation of specific sites, providing useful tools for constructing mtDNA mutation models for mitochondrial disease research. In zebrafish, the DdCBE editing efficiency of mtDNA can reach 88.32% via embryo microinjection in the F0 generation.^166^ Another editing tool, FusX TALE Base Editor (FusXTBE), with an efficient editing site design script based on Python, induced 90+% editing efficiency in zebrafish.^451^ In mammalian models, the editing efficiency is much lower. The editing efficiency of DdCBE in F0 rats was reported to be 36.33%,^162^ and the initial mtDNA editing efficiency of DdCBE via embryo microinjection in mice was reported to be 0.25–23%.^170^ Some strategies have been developed to enhance this efficiency. DdCBE combined with a nuclear export signal (DdCBE-NES) enhances mtDNA editing by 38.9%.^452^ Coinjecting mitoTALENs with DdCBEs or DdCBE-NES yielded a 1.7–3-fold increase in editing efficiency.^452^

### Application of artificial mitochondrial transfer

#### Mitochondrial transplantation

##### Preclinic studies

Most preclinical studies involve directly injecting mitochondria into the target organ or tissue, including the muscles,^453,454^ cardium,^455,456^ dorsal root ganglion,^457^ spinal cord,^372^ cerebral cortex,^458^ vitreous cavity of the eyes,^459^ internal carotid artery,^324^ left coronary ostium,^458^ renal capsule,^460^ lateral ventricles,^461,462^ striatum,^463^ femoral artery,^463^ ex vivo lung perfusion (EVLP),^464^ medial forebrain bundle (MFB),^406^ and renal artery.^465^ A few systematic administrations, including intravenous injection, have also achieved satisfactory results.^466–470^ Shi et al. conducted an intravenous injection of isolated mitochondria in mice and observed the distribution of exogenous mitochondria in multiple organs.^470^ Nakamura et al. reported that intravenously administered mitochondria can traverse the blood-brain barrier and disperse throughout the brain.^469^

In addition to the above direct administration, mitochondria can also exert their effects by being loaded in BMSCs,^365,471^ human peripheral blood mononuclear cells,^472^ and EVs.^395^ The details of preclinical studies on mitochondrial transplantation are summarized in Table 4.

**Table 4:** Preclinical studies on mitochondrial transplantation

Author	Animal model	Mitochondria source	Transplantation method	(Implied) mitochondria receptor	Mitochondria amount in per animal model	Main result
Musculoskeletal Regeneration						
Kim^453^	Rat muscular atrophy (via DEX)	Human UC-MSCs	Intramuscular injection	Muscle tissue	0.5 µg or 5 µg mitochondria	Increased muscle mass by 1.5-fold and decreased lactate concentration by 2.5-fold at 1 week
Alway^466^	Mouse muscle injury (via BaCl_2_)	Donor mouse liver	IV	Muscle tissue	50 μg mitochondria	Enhanced the rate of muscle regeneration and restoration of muscle function following injury
Zeng^454^	Mouse limb ischemia (limb ligation)	Human UC-MSCs	Intramuscular injection	-	1 × 10^7^ mitochondria	Increased muscle energy and adipocyte browning, which promotes the recovery of motor function
Guo^471^	Rat cranial defect	Donor rat BMSCs	Transplant Mt-rec BMSCs (obtained via centrifugation in vitro)	-	-	Increased new bone formation
Lin^79^	Mouse limb ischemia model	ADSCs	Transplant Mt-rec ECs (obtained via coculture in vitro)	-	-	Artificial transplantation of exogenous mitochondria can enhance the ability of human ECs to engraft and revascularize ischemic tissues
Myocardial protection						
Liang^455^	Mouse RI (LAD occlusion)	Human BMSCs	Intramyocardial injection	Endothelial cells	8 × 10^5^ mitochondria	Protecting cardiac function
Ikeda^473^	Mouse RI (LAD occlusion)	Cardiomyocytes derived from iPSc	Intramyocardial injection	Cardiomyocytes	1 × 10^8^ EVs	Restored bioenergetics and mitochondrial biogenesis; significantly improved post-myocardial-infarction cardiac function
McCully^474^	Rabbits RI (LAD occlusion)	Donor rabbit ventricular	Intramyocardial injection	Myocardium	6 × 10^6^ mitochondria	Significantly enhanced postischemic functional recovery and cellular viability.
Blitzer^458^	Pigs RI (LAD occlusion)	Autologous pectoralis	Injection into the left coronary ostium	-	1 × 10^9^ mitochondria	Decreased myocardial infarct size; increased regional and global myocardial function
Masuzawa^456^	Rabbits RI (LAD occlusion)	Autologous pectoralis	Intramyocardial injection	Cardiomyocytes	8 × 10^6^ mitochondria	Protected the heart from ischemia-reperfusion injury
Kaza^475^	Pigs RI (circumflex artery occlusion)	Autologous pectoralis	Intramyocardial injection	Myocardium	1 × 10^7^ mitochondria	Significantly enhanced myocardial cell viability
Neuroprotection						
Huang^457^	Rats TNP (nerve ligation)	Donor soleus	Ipsilateral L5 DRG microinjections	Spinal cord and sciatic nerve	100 µg mitochondria	Mitigated apoptosis and neuroinflammation
Li^372^	Rats SCI (spinal cord hit).	Rats BMSCs	Injected into the injured spinal cord	Neurons	-	Improved locomotor functional recovery
Xu^467^	Mouse SCI (modified Allen’s percussion)	BMDMs	IV	Macrophages	1–2 × 10^6^ compound	Promoted tissue regeneration and bolstered functional recovery
Jiang^459^	Mouse RGC degeneration (Ndufs4 knockout)	Human iPSC-MSCs	Transplant iPSC-MSCs (spontaneous transfer in vivo)	RGC	-	Significantly increased RGC survival and improved retinal function
Xie^324^	Rats stroke model (MCAO)	Neuro-2a cell	Injected into the internal carotid artery	-	180–200 μg protein	Improved neurobehavioral deficits, and reduced infarct size
Zhang^461^	Rats stroke model (MCAO)	Autologous pectoralis	Infused into the lateral ventricles	Neurons	5 ×10^6^ mitochondria	Decreased brain infarct volume and reversed neurological deficits
Huang^463^	Rats stroke model (MCAO)	BHK-21 cells	Infused into the ischemic striatum or the femoral artery	Neurons, astrocytes, and microglia	750 μg mitochondria	Attenuated brain infarct area and neuronal cell death
Pourmohammadi-Bejarpasi^462^	Rats stroke model (MCAO)	Human UC-MSCs	Injected into the lateral ventricle	-	-	Decreased astrogliosis and microglia activation, reduced infarct size, and improved motor function
Nakamura^469^	Mouse stroke model (MCAO)	Mouse placenta	IV	-	100 μg protein	Significantly decreased infarction
Babenko^476^	Rat stroke model (MCAO)	hBMSCs	Transplant hBMSCs pre-cocultured with neurons (spontaneous transfer in vivo)	Neurons	-	Compared with native hBMSCs, pre-cocultured hBMSCs demonstrated a more pronounced reduction in neurological deficits
Zhao^478^	mice TBI model (by CCI)	Allogeneic liver, and autogeneic muscle	Injected into cerebral cortex	neurons, astrocytes, and microglia	1 × 10^6^ mitochondria	Effectively improve mice’s spatial memory and cognitive function following TBI
Chang^406^	Rat PD (via 6-OHD)	PC12 cells or human osteosarcoma cybrids	Injected into the MFB	Dopaminergic neurons of SNc	1 μg mitochondria	Improved the locomotive activity in the PD rats
Shi^470^	Mouse PD (via MPTP)	HepG2 cells	IV	-	0.5 mg/kg mitochondria	Increased the activity of electron transport chain; decreased ROS; prevented cell apoptosis and necrosis.
Nitzan^468^	Mouse AD (via amyloid-β)	Hela cell	IV	-	200 μg mitochondria	A significantly better cognitive performance
Respiratory Protection						
Islam^365^	Mouse ALI (via LPS)	Mouse BMSCs	Transplant BMSCs (spontaneous transfer in vivo)	Alveolar epithelium	-	Protected against ALI by restituting alveolar bioenergetics
Morrison^395^	Mouse ALI (via LPS)	Human MSCs	Transplant Mt-rec AMs (obtained via coculture in vitro)	AMs	-	Decreased inflammation and lung injury
Johnatas Dutra^479^	Mouse ALI (via LPS)	Human MSCs	Systemic administration	HSAECs and HPMECs	-	Improved alveolar-capillary barrier properties through restoration of mitochondrial functions
Ahmad^480^	Mouse AAI (via Rot)	MSCs	Transplant MSCs (spontaneous transfer in vivo)	ECs	-	Overexpression of Miro1 in MSC is associated with greater mitochondrial donation, greater rescue of injured ECs
Cloer^464^	Porcine and human ex-vivo ischemia-reperfusion lung	Porcine ventricle	Ex-vivo lung perfusion	ECs	-	Decreased tissue oxidative and inflammatory signaling; improved lung function
hepatic protection						
Lu^512^	Mouse liver IRI model (hepatic artery and portal vein occlusion)	Human UC-MSCs	IV	neutrophils		Restored the mitochondrial status and functions in neutrophils to reduce neutrophil extracellular traps formation.
Immunomodulatory						
Court^472^	Humanized xenogenic‐GVHD mouse	Human UC‐MSC	Transplant Mt-rec human PBMCs (obtained via Mitoception in vitro)	-	-	Significantly improved survival; decreased tissue damage and organ T cell infiltration
Renal protection						
Yuan^482^	Mouse DM (via STZ)	Mouse MSCs	Transplant MSCs (spontaneous transfer in vivo)	Macrophages	-	Inhibited the inflammatory response and alleviated kidney injury.
Konari^460^	Rats DM (via STZ)	Rats MSCs	Injected under the renal capsule	ECs	-	Improved the cellular morphology of proximal tubular ECs, tubular basement membrane and brush border
Doulamis^465^	Pigs AKI (renal arteries occlusion)	Autologous SCM	Intra renal arterial injection	ECs	1 × 10^9^ mitochondria	Significantly enhanced renal function and reduced renal damage.
Jabbari^483^	Rat AKI (via kidney hilum occlusion)	Autologous pectoralis	Intrarenal arterial injection	Renal cells	3 × 10^6^ mitochondria	Prevented renal tubular cell death; restored renal function; ameliorated kidney damage; improved regenerative potential of renal tubules
Cancer						
Ippolito^351^	Immunodeficient mouse xenograft	Human CAFs	Spontaneous transfer	Human PCa cells	-	Enhanced motile features and lactate metabolism of PCa cells
Moschoi^353^	Immunodeficient mouse xenograft	Bone marrow	Spontaneous transfer	AML cells	-	Provided a clear survival advantage following chemotherapy
Burt^354^	Immunodeficient mouse xenograft	Activated MSCs/CAFs	Spontaneous transfer	AML cells	-	Prevented cell death from ROS-inducing chemotherapy

*6-OHD* 6-hydroxydopamine, *AAI* allergic airway inflammation, *AD* Alzheimer’s disease, *ADSCs* Adipose-derived stem cells, *AKI* Acute kidney injury, *ALI* Acute lung injury, *AML* Acute myeloid leukemia, *AMs* Alveolar macrophages, *BMDMs* bone marrow-derived macrophages, *BMSCs* bone marrow stromal cells, *CAFs* cancer-associated fibroblasts, *DEX* dexamethasone, *DM* diabetes mellitus, *DRG* dorsal root ganglion, *ECs* endothelial cells, *EVs* extracellular vesicles, *GVHD* graft-versus-host disease, *HPMECs* human pulmonary microvascular endothelial cells, *HSAECs* human small airway endothelial cells, *iPSc* induced pluripotent stem cell-derived, *IRI* ischemia-reperfusion injury, *IV* intravenous, *LAD* left anterior descending (artery), *LPS* lipopolysaccharide, *MCAO* middle cerebral artery occlusion, *MFB* medial forebrain bundle, *MI* myocardial infarction, *MPTP* 1-methyl-4-phenyl-1,2,3,6-tetrahydropyridine, *MSCs* mesenchymal stem cells, *PBMCs* peripheral blood mononuclear cells, *PCa* prostate cancer, *PD* Parkinson’s disease, *RGC* retinal ganglion cell, *RI* regional ischemia, *ROS* reactive oxygen species, *Rot* rotenone, *SCI* spinal cord injury, *SCM* sternocleidomastoid muscle, *SNc* substantia nigra pars compacta, *STZ* streptozotocin, *TBI* traumatic brain injury, *TNP* traumatic neuropathic pain, *UC-MSCs* umbilical cord mesenchymal stem cells

**Musculoskeletal system**. BMSCs containing extra mitochondria from donor rats increased OXPHOS activity, ATP production, and bone repair in cranial defect model rats.^471^ Mitochondria from hUCMSCs promoted the recovery of motor function in the gastrocnemius muscle of acute limb Ischemia-reperfusion injury (IRI) model mice by increasing adipocyte browning and ATP production and reducing apoptosis.^454^ Tail vein injection of mitochondria isolated from donor mouse livers accelerated muscle repaired and restored muscular function in a muscle injury mouse model,^466^ and similar results have also been reported in a muscular atrophy rat model.^453^ Notably, recent research revealed an attractive result: the new autologous-produced mitochondria stimulated by the degradation process of transplanted mitochondria in ECs, rather than the transplanted mitochondria, enhanced metabolic activity and vessel formation.^79,80^

**Circulation system**. Myocardial ischemia in mice,^455,473^ rabbits,^456,474^ and pigs^458,475^ is improved by local mitochondrial injection. The related mechanism may include reducing cell apoptosis, ROS, endothelial cell senescence, and fibrosis and promoting ATP production, endothelial cell proliferation, and angiogenesis. Although most transplanted mitochondria were isolated from stem cells, autologous pectoralis could also provide optimal mitochondria without affecting the immune response or mtDNA heterogeneity. Most preclinical studies have involved directly injecting mitochondria into the myocardium, while Blitzer^458^ injected mitochondria into the left coronary ostium. Considering that coronary angiography techniques are quite mature in the clinic, mitochondrial transplantation via coronary arteries might be practical and potentially useful.

**Neural system**. *Cerebral injury:* Similar to myocardial ischemia, mitochondrial transplantation has been applied in cerebral ischemia mainly to enhance cellular functioning by decreasing ROS levels and regulating cellular metabolism. Cerebral ischemia models in both rats^324,461–463,476^ and mice^469^ have been confirmed to improve cognitive function and motor function and reduce infarct size and neurobehavioral deficits. However, whether mitochondria can pass through the blood-brain barrier is controversial. Xie et al.^324^ reported that no mitochondria pass through the intact blood-brain barrier, while Shi et al.^470^ and Nakamura et al.^469^ observed mitochondria in the brain after intravenous injection. One potential explanation is that disruption of the blood-brain barrier may occur during stroke,^477^ allowing the penetration of mitochondria. From this perspective, Dave et al. suggested a synergistic approach including mitochondria and HSP27 to enhance the viability of brain endothelial cells and enhance the structural stability of tight junctions.^394^ In addition to cerebral ischemia, injection of mitochondria into the cerebral cortex of traumatic brain injury (TBI) model mice improved spatial memory and alleviated anxiety.^478^ Notably, the findings obtained in vitro demonstrate the facile internalization of foreign mitochondria by neurons, astrocytes, and microglia,^478^ suggesting that mitochondrial transplantation might exert effects via multiple pathways.

*Spinal Cord Injury:* Mitochondrial transplantation into spinal cord injury (SCI) rat^372,457^ and mouse^467^ models mitigates apoptosis and neuroinflammation, improves locomotor functional recovery, and promotes tissue regeneration. An engineered mitochondrial compound (mitochondria-Tpp-CAQK-FAM) has been designed.^467^ Tpp are used to bind mitochondria via charge attraction. The CAQK peptide can efficiently bind to proteoglycan molecules in the damaged region after SCI, and FAM fluorescent molecules can be integrated for real-time monitoring. Transplanted mitochondria isolated from mouse bone marrow-differentiated macrophages were absorbed by recipient macrophages in an SCI model and effectively modulated inflammation. The ability to regulate and target inflammation shows significant potential for SCI management.

*Neural degeneration:* In Parkinson’s disease (PD) mice^470^ and rats,^406^ mitochondrial transplantation decreased ROS levels, prevented cell apoptosis and necrosis, and improved locomotive activity. In AD model mice, mitochondrial transplantation significantly improved cognitive performance.^468^ In addition to the central nervous system, peripheral nerve degeneration can be alleviated by mitochondrial transplantation. Jiang^459^ injected mitochondria from human induced pluripotent stem cells (iPSC-MSCs) into the vitreous cavity of the eyes of a retinal ganglion cell (RGC) degeneration model and showed a significant increase in RGC survival and retinal function.

**Respiratory system**. Islam et al. used live optical tools and observed that intratracheally instilled mBMSCs transferred mitochondria to the alveolar epithelium, leading to a protective effect.^365^ Subsequently, mitochondrial transplantation via lipopolysaccharide (LPS) was explored in mouse acute lung injury models, and reduced inflammation and lung injury were observed.^395,479^ In addition, rotenone (Rot)-induced asthma in a mouse model was alleviated by mitochondrial transplantation, resulting in the rescue of injured epithelial cells and a reduction in epithelial-mediated amplification of the immune response.^480^ Using mitochondria via EVLP to preserve lungs for transplantation could also be beneficial. Cloer et al. reported that mitochondrial transplantation reduced resistance and inflammation in both porcine and human ex vivo lungs, potentially reducing graft rejection.^464^

**Immune system**. Although the details of signal transduction between transplanted mitochondria and immune cells remain unknown, the biological function of immunocompetent cells is directly linked to their energetic status, which is influenced by mitochondria.^481^ Transplantation of mitochondria from human umbilical cord-derived mesenchymal stem cells (UC-MSCs) to human peripheral blood mononuclear cells (PBMCs) induced Treg differentiation in vitro.^472^ Administering mitochondria-treated T cells in a graft-versus-host disease (GVHD) mouse model greatly enhanced survival and decreased the invasion of specific T cells, revealing that mitochondrial transplantation has substantial potential for T‐cell reprogramming. Macrophages are another type of target cell for mitochondrial transplantation. Morrison et al. transplanted human MSC-derived mitochondria into alveolar macrophages (AMs) in vitro, and mitochondria-containing AMs significantly reduced inflammation in acute lung injury model mice.^395^ Xu et al. transplanted mitochondria from mouse bone marrow-derived macrophages (BMDMs) into the SCI environment to modulate inflammation via absorption by recipient macrophages.^467^

**Urinary system**. Chronic kidney injury related to diabetes mellitus in mouse^482^ and rat^460^ models was alleviated by mitochondrial transplantation by switching Macrophages from M1 to M2, inhibiting the inflammatory response and improving the cellular morphology of renal proximal tubular epithelial cells and the architecture of the basement membrane. In addition, acute kidney injury in rat^483^ and porcine^465^ models was alleviated by transplanting autologous mitochondria via renal arterial injection.

Notably, the administered mitochondria in swine were promptly absorbed by the kidneys and were not detected in any other organs, highlighting the efficiency and safety of intrarenal arterial mitochondria injection in treating kidney impairment.

**Cancer**. As stated above, the effects of transferred mitochondria in cancers remain controversial. No therapeutic application of mitochondrial transplantation has been applied for cancer treatment in vivo. Only limited pilot studies have been conducted on the behavior of transplanted mitochondria related to cancer cells in vivo. The aggressiveness of prostate cancer cells (PC3 and DU145) in vivo is enhanced when they are coinjected with cancer-associated fibroblasts capable of providing mitochondria.^351^ Similarly, AML-derived cells acquire normal mitochondria from coinjected BMSCs, increasing survival times after chemotherapy.^353^ Coinjected MSCs also transport mitochondria to rescue ALL B-cell precursors from ROS-inducing chemotherapy.^354^ Given the in vivo findings, blocking mitochondrial transfer rather than transplanting mitochondria might be beneficial in cancer therapy.

##### Clinical studies

Emani et al. injected mitochondria from autologous nonischemic skeletal muscle into 5 children requiring central extracorporeal membrane oxygenation (ECMO) assistance due to myocardial dysfunction following heart surgery.^484^ Four patients were successfully withdrawn from ECMO assistance. Guariento et al. conducted a comparative study (MT group, *n* = 10; control group, *n* = 14) with similar settings given these encouraging results.^485^ The rates of successful separation from ECMO (80% vs. 29%), median time to functional recovery after revascularization (2 days vs. 9 days), and incidence of cardiovascular events (20% vs. 79%) were significantly greater in the mitochondrial transduction group.

Jacoby et al. intravenously infused autogenous CD34^+^ cells augmented ex vivo (hematopoietic stem cells) that received mitochondria from maternal PBMCs into six patients with single large-scale mitochondrial DNA deletion syndrome (SLSMD).^486^ The technique was well tolerated, and all major adverse events during the study were related to the leukapheresis process or the underlying disease at the beginning of the study. After mitochondrial transplantation, all 6 patients exhibited an elevation in mtDNA content in their peripheral blood cells, ranging from 6 to 12 months, compared to the initial measurements. Two patients had a notable enhancement in their aerobic capabilities.

Considering the substantial potential of mitochondrial transplantation, several clinical trials, including those involving cerebral ischemia (NCT04998357), myocardial ischemia/reperfusion injury (NCT05669144), ECMO (NCT02851758), refractory polymyositis or dermatomyositis (NCT04976140), are ongoing. These trials might lead to a breakthrough in the application of mitochondrial transplantation (Table 5).

**Table 5:** Clinical trials of engineered mitochondria in disease treatment

Study	Population	Sample size	Mitochondrial interventions	Administration route	Amount	main Result
Published research						
Yu-Wai-Man^323^ NCT026527080	LHON subjects carrying the *m.11778G* > *A (MT-ND4)* mutation	37	rAAV2/2-*ND4*	Intravitreal injection	9 × 10^10^ vg	68% of subjects had a clinically relevant recovery in BCVA in at least one eye, and 78% had an improvement in vision in both eyes.
Liu^513^ NCT03153293	Patients with LHON and an *MTND4m.11778G* > *A* mutation	149	AAV2-*ND4*	Intravitreal injection	5 × 10^11^ vg	Significant improvement in visual acuity was seen within 3 days of treatment, these improvements remained stable in 12-month follow-ups.
Yang^514^ NCT01267422	Patients with LHON	9	rAAV2-*ND4*	Intravitreal injection	5 × 10^9^ vg or 1 × 10^10^ vg	Visual function improvement was observed in both treated eyes and untreated eyes
Yuan^515^ NCT01267422	patients with the LHON *mt11778G*→*A* mutation	9	Injection of recombinant Adeno-Associated Virus-NADH dehydrogenase, subunit 4 (complex I)	Intravitreal injection	5 × 10^9^ vg or 1 × 10^10^ vg	Most patients experienced significant improvements in BCVA after treatment, with varying degrees of sustained or transient improvements over time.
Guy^516^	patients with visual loss and mutated *G11778A* mitochondrial DNA	14	Injection with the gene therapy vector AAV2(Y444,500,703F)-*P1ND4v2* into one eye	Intravitreal injection	5 × 10^9^ vg or 2 × 10^10^ vg	This allotopic gene therapy for LHON at low and medium doses appears safe and does not damage the temporal peripapillary retinal nerve fiber layer
Emani^484^	Pediatric patients requiring postcardiotomy ECMO	5	Transplant mitochondria from autologous skeletal muscle	Direct intramyocardial injection	1 × 10^8^ mitochondria	4 in 5 subjects demonstrated ventricular function improvement and separated from ECMO support.
Guariento^485^	pediatric patients requiring postcardiotomy ECMO.	MT, *n* = 10; Control, *n* = 14	Transplant mitochondria from autologous rectus abdominis muscle	Direct epicardial injection	1–10 × 10^8^ mitochondria	The ratio of separation from ECMO in MT group is higher (80% VS 29%, P = 0.02)
Jacoby^486^	SLSMDs	6	MAT technology	Intravenously infusion	CD34+ cells were incubated with about 1 mU mitochondria	MAT decreased mtDNA heteroplasmy, and improved muscle strength and endurance in two individuals
Ongoing research						
NCT04998357	Cerebral ischemia	20	Transplant mitochondria from autologous muscle	Injection into cerebral vessels	-	-
NCT04976140	Refractory polymyositis or dermatomyositis	9	Transplant HUCSCs-derived mitochondria	Intravenous injection	-	-
NCT05669144	Patient candidate for CABG surgery	(MT, *n* = 5; Control, *n* = 5)	Transplant mitochondria from autologous pectoralis muscles	Intracoronary and intra-myocardial injection	-	-
NCT02851758	Pediatric cardiology patients on ECMO	16	Transplant mitochondria from autologous skeletal muscle	Direct injection into the myocardium	-	-
NCT02652767/NCT02652780/NCT03293524	LHON with *G11778A* mutation in the mitochondrial *ND4* Gene	39/37/90	rAAV2/2-*ND4*	Intravitreal injection	-	-

*AAV* adeno-associated viruses, *BCVA* best corrected visual acuity, *CABG* coronary artery bypass grafting, *ECMO* extracorporeal membrane oxygenation, *HUCSCs* human umbilical cord mesenchymal stem cells, *IRI* ischemia-reperfusion injury, *LHON* Leber hereditary optic neuropathy, *logMAR* logarithm of the minimum angle of resolution, *MAT* mitochondrial augmentation therapy (transplant autologous CD34+ cells those had received mitochondria from maternally PBMCs ex vivo), *MTS* mitochondrial targeting sequence, *PBMCs* peripheral blood mononuclear cells, *SLSMDS* single large-scale mitochondrial DNA deletion syndromes

To date the primary artificial mitochondrial transfer techniques applied in clinical trials for mitochondrial diseases or mitochondrial-related disorders involve direct injection or ex vivo coculture. Most of these techniques remain in the laboratory or preclinical research stages. Nevertheless, their rapid progress is highly encouraging. For research purposes, these technologies provide precise, quantifiable, and highly reproducible methods for mitochondrial transplantation, enabling the exploration of post-transplant mitochondrial behavior and mechanisms. From a clinical application perspective, the advancement in miniaturization and non-invasiveness of transfer tools, along with improvements in the safety and targeting of surface modifications, offer promising prospects for future clinical applications. Additionally, the adoption of indirect transplantation strategies may facilitate the rapid clinical translation of many techniques. This approach involves extracting patient cells for ex vivo expansion and mitochondrial transfer before returning them to the patient. Such a method could significantly broaden the application of mitochondrial transfer techniques, achieving almost the same extensive applicability as cellular therapies.

#### Mitochondrial replacement therapy (MRT)

MRT was initially conceived to address infertility in older women and subsequently refined to prevent mitochondrial diseases, influencing a large population.^487,488^ At present, the available options for a couple to prevent the spread of mitochondrial problems are minimal. These include oocyte or embryo donation and the termination of a pregnancy upon prenatal diagnosis.

Mitochondria play a vital role in maintaining the quality of oocytes, promoting normal fertilization, sustaining subsequent embryo formation via energy generation, maintaining Ca^2+^ balance, and regulating oxidative stress.^489^ Mitochondrial manipulation has emerged as a new approach for enhancing the effectiveness of in vitro fertilization therapies in women with oocyte quality issues.^490^

MRTs involve two primary methodologies: heterologous and autologous. In the heterologous technique, mitochondria are obtained from an exogenous source. Heterologous procedures involve transferring the cytoplasm to the recipient oocyte or, vice versa, transferring the nucleus. Ooplasmic transfer (OT), also called partial cytoplasm transplantation, was the first technique developed to address defects in oocyte quality.^491^ OT involves transplanting cytoplasm components from a donor’s oocyte to the recipient oocyte, which contains functional mitochondria.^492^ The first pregnancy involving OT was announced in 1997.^73^ Conversely, total cytoplasm transplantation transports genetic material from a defective oocyte or zygote to a healthy cytoplasm, which can be achieved with various cytoplasm providers, including germinal vesicles (GVs),^493^ spindles,^494,495^ pronuclei,^496^ polar bodies,^497,498^ and blastomeres.^499^ However, the issue of mitochondrial heteroplasmy remains unresolved and leads to ethical conflicts, such as ‘three-parent’ babies.^500^

The autologous technique effectively solves the ethical and safety concerns related to heteroplasmy. Alternative sources of autologous mitochondria have been widely researched.^500^ Suggested autologous mitochondria sources include undeveloped oocytes,^124^ granulosa cells,^501^ germline stem cells (GSCs),^502^ and other stem cells.^503^

Due to the large difference between germ cell mitochondrial replacement and somatic cell mitochondrial transplantation, this review focused mainly on somatic cell mitochondrial transplantation. Detailed information on germ cell mitochondrial replacement has been reviewed.^490,500,504,505^

## Conclusions and perspectives

Over the past decade, there have been significant advances in engineered mitochondria, including new mitochondrial gene editing tools and artificial mitochondrial transfer platforms. Comprehensive knowledge of the pathogenic genes, mitochondrial subcellular distribution, and the toxicity of therapeutic vectors is essential for the successful clinical implementation of these therapies. Appropriate cellular and animal models related to mitochondrial disease and precise biomarkers are necessary for designing effective trials.

Mitochondrial gene editing faces several significant challenges, with one of the most pressing issues being in vivo delivery and off-target effects. One promising direction that current research is actively exploring is the development of highly efficient targeted delivery systems for editing tools. Enhancing the specificity and effectiveness of these delivery systems could significantly improve the precision of mitochondrial gene editing in living organisms. An alternative direction that circumvents delivery issues is to manipulate reproductive cells or embryos to prevent the transmission of genetic disorders from the outset. In these scenarios, optimizing editing tools based on the precise identification of mutations using the latest detection technologies, such as modified mitochondrial genome sequencing,^506,507^ is crucial.

Regarding mitochondrial transplantation, a major limitation is its current applicability primarily to conditions characterized by “inadequate normal mitochondria,” rather than “excessive abnormal mitochondria.” Research into enhancing the clearance of abnormal mitochondria through mechanisms such as mitophagy and mitocytosis may address this gap. Additionally, bidirectional mitochondrial transfer^329,508^—a spontaneous phenomenon observed in several studies—presents another potential solution. Understanding and regulating the direction and process of mitochondrial transfer could be a crucial element in optimizing engineered mitochondria applications. Therefore, further research relying on advancements in tracing technologies is needed to understand mitochondrial transfer better. Long-term in vivo observation of transplanted mitochondria will provide valuable insights into their behavior and fate.

Future widespread clinical applications of mitochondrial gene editing are likely to be first realized in scenarios that are relatively easier to manipulate and observe. These include interventions in the reproductive cells or zygotes of parents with hereditary mitochondrial diseases or causative genes, and in mitochondrial diseases mainly affecting single or limited organs, such as LHON. In these cases, engineered mitochondria could be confined to a specific area, improving targeting and delivery effectiveness. As for mitochondrial transplantation, autologous resources might be first adopted for their reliable safety. Insufficient mitochondrial functions in the tissues that are supposed to have highly active mitochondria, such as myocardial insufficiency and neurological dysfunction, are optimal indications. Given the rapid and continuous technological advancements in engineered mitochondria through both gene editing and artificial mitochondrial transfer, their potential application is expected to continually expand across a wide range of diseases, from genetic mitochondrial diseases to non-genetic scenarios. Furthermore, a potentially more readily implementable approach is to perform ex vivo editing on mitochondria, followed by the selection and transfer of successfully edited mitochondria into the target organ. This method may simultaneously address specific challenges such as poor in vivo operability and the introduction of heterogeneity, thereby broadening the application boundaries.
